# Analysis and dynamical structure of glucose insulin glucagon system with Mittage-Leffler kernel for type I diabetes mellitus

**DOI:** 10.1038/s41598-024-58132-5

**Published:** 2024-04-05

**Authors:** Maryam Batool, Muhammad Farman, Abdul Sattar Ghaffari, Kottakkaran Sooppy Nisar, Shankar Rao Munjam

**Affiliations:** 1 Institute of Mathematics, Khawaja Fareed University of Engineering and Information Technology, Rahim Yar Khan, Pakistan; 2 Department of Computer Science and Mathematics, Lebanese American University, Beirut, Lebanon; 3 Department of Mathematics, College of Science and Humanities in Alkharj, Prince Sattam Bin Abdulaziz University, Alkharj, 11942 Saudi Arabia; 4 School of Technology, Woxsen University, Hyderabad, Telangana 502345 India

**Keywords:** Mittage-Leffler Kernel, Boundedness, Uniqueness, Lyapunov Stability, GIG system, Applied mathematics, Physiology

## Abstract

In this paper, we propose a fractional-order mathematical model to explain the role of glucagon in maintaining the glucose level in the human body by using a generalised form of a fractal fractional operator. The existence, boundedness, and positivity of the results are constructed by fixed point theory and the Lipschitz condition for the biological feasibility of the system. Also, global stability analysis with Lyapunov’s first derivative functions is treated. Numerical simulations for fractional-order systems are derived with the help of Lagrange interpolation under the Mittage-Leffler kernel. Results are derived for normal and type 1 diabetes at different initial conditions, which support the theoretical observations. These results play an important role in the glucose-insulin-glucagon system in the sense of a closed-loop design, which is helpful for the development of artificial pancreas to control diabetes in society.

## Introduction

Specifically, blood sugar levels are regulated by two hormones that have antagonistic effects on the human body. In contrast to insulin, which promotes the uptake of blood sugar by muscles and adipose tissues and stores it as glycogen in the liver, glucagon is secreted to treat hypoglycemia following fasting or meals without carbohydrates, but insulin is released to prevent blood sugar levels from going above a specific threshold after meals. Because of this, it will be challenging to control glycemia whenever glucagon or insulin secretion is disrupted. Research on $\alpha$-cells and glucagon is less important than that on $\beta$-cells and insulin, despite the significance glucagon plays in the regulation of blood sugar^1^. According to studies, type 2 diabetic participants do not display the same glucagon suppression in response to the glucose stimulus as healthy people. Plasma glucagon levels significantly decrease, plasma insulin levels significantly rise, and as a result, plasma glucose levels are normal in non-diabetic individuals. The opposite is true for those with type 2 diabetes, who also have post-prandial hyperglycemia and low plasma insulin levels that are at or above pre-prandial levels^2^. According to the bi-hormonal hypothesis proposed by Unger and Orci in 1975, hyperglycemia is caused by both an excess of glucagon and insulin insufficiency or resistance, which cause the liver to create more glucose than is needed for the utilization of the glucose, leading to diabetes^3^. In contrast to $\alpha$-cells and glucagon, mathematical models of the dynamics of glucose, insulin, and $\beta$-cells have recently drawn a lot of attention^4–7^. In this study, we provided a mathematical model of the coupled dynamics of glucose, insulin, $\alpha$ and $\beta$ cells, and glucagon^8^.

Atangana^9^ has proposed an entirely novel technique for fractional calculus called the fractal fractional derivative. The idea behind this subject is frequently quite helpful for solving some difficult issues. Two orders of the operator are the fractal dimension and the fractional order. The conventional method is outperformed by this new approach to fractal fractions^10–12^. This is so that one can simultaneously study fraction operators and fractal dimensions while dealing with fractal fractional derivatives. The huge advantage of this operator is that it enables you to construct models that more precisely describe systems with memory effects. In addition, there are other real-world concerns that call for knowledge of a system’s information capacity. utilised unique applications and a variety of kernels to report some breakthroughs in fractal-fractional differential equations^13,14^. Fractional calculus is parqueting the interest of scientists all around the world because of its many benefits and useful applications in physics and engineering. Hereditary traits, memory, and crossover behaviour can only be observed using a model with a fractional-order system^15–17^. The behaviour of a fractional-order diabetes model was examined in the research paper. The fractional order derivative of the glucose concentration was taken into account along with the interactions between glucose, insulin, and glucagon. The model’s equilibrium points were examined for stability, and the scientists also looked at how the fractional order derivative affected the dynamics of the system. The outcomes demonstrated that the model’s behaviour could be influenced by the fractional order derivative, producing dynamics that were more complex than in the traditional integer order scenario^18^. The literature on fractional order modelling and analysis of diabetes was thoroughly reviewed by them. The authors explored a range of modelling techniques for diabetes, such as conventional integer order models, fractional order models, and hybrid models. They emphasised the benefits of employing fractional order models, including their capacity to reproduce memory and non-locality effects observed in biological systems^19^. Researchers have been examining the intricate interactions between glucose-insulin dynamics, metabolic regulation, and patient behaviour in the study of behavioral dynamics in the fractional order diabetes model in recent years^20^. Researchers have been able to capture the non-integer order derivatives that result from patient behavior by introducing fractional calculus into diabetes modelling, providing a more accurate picture of the dynamics of the disease^21^. The existing fractional order models for diabetes and their uses in evaluating the course of the disease, the effectiveness of insulin treatment, and glucose control were also reviewed by some writers^22^. Nevertheless, conventional or classical fractional-order models find it challenging to effectively articulate the concept of piecewise fractional-order derivatives to get over this limitation due to the complexity of the epidemic, particularly during crossover periods. With this approach, we want to provide a more realistic picture of the intricate crossover behaviours present in the dynamics of the pandemic and some real life application through different kinds of fractional order derivative^28–32^.

Fractional calculus has a strong history and plays a major role in the simulation of physical phenomenon of real life. Recently, there has been a lot of interest in the study of fractional calculus. Fractional calculus and fractional processes have become one of the most useful approaches to deal with a variety of problems in applied sciences due to memory and hereditary properties. A number of studies for fractional order linear, non-linear and complex dynamical mathematical models have been presented with interesting results during recent years. Therefore, compared to the traditional integer order models, fractional order models seem to be more objective and flexible. There is a long history of mathematical modelling on the topic of glucose metabolism. There are various reasons for using models. Models have been used to infer physiologically significant factors from experimental data in an indirect manner, to provide a clear quantitative depiction of pathophysiological pathways, and to derive clinically valuable indexes from basic experimental procedures. As the impact of type 1 diabetes on society grows, models related to the disruption of the glucose homeostasis system are being developed and utilised more frequently. Currently, diabetes mellitus is one of the major issues all over the world. We construct a fractional-order glucose-insulin-glucagon system with a novel fractional technique. For this purpose, Section “Introduction” is an introduction, and Section “Basic concepts has some useful definitions”. A mathematical model with a description is presented in Section “Diabetes model with fractional derivative”. An analysis with different aspects and stability is given in Section “Analysis of proposed model”. The advanced numerical scheme fractal fractional is present in Section “Computational analysis with fractal fractional operator for reliable solutions”. A numerical simulation is given in Section “Numerical simulation and discussion” to see its physical interpretation and conclusion in Section “Conclusion”.

## Basic concepts

### Definition 1

^23,24^ For a power law kernel in the Riemann–Liouville sense is given as;

1 $$\begin{aligned} _0^{FFP}D_t^{\alpha ,\eta } x(t) = \frac{1}{\Gamma (1- \alpha )} \frac{d}{dt^\eta } \int _0^t (t-\varsigma )^{- \alpha } x(\varsigma ) d\varsigma \end{aligned}$$

with $$0 \le \alpha , \eta \le 1$$. Where

$$\begin{aligned} \frac{df(t)}{dt^\eta } = \lim _{t\rightarrow t_1} \frac{f(t) - f(t_1)}{t^{2-\eta }-t_1^{2-\eta }}(2-\eta ) \end{aligned}$$

The corresponding power law kernel fractal-fractional integral of order $(\alpha ,\eta )$ is given as

2 $$\begin{aligned} _0^{FFP}I_t^{\alpha ,\eta } x(t) = \frac{1}{\Gamma (\alpha )}\int _0^t (t - \varsigma )^{\alpha - 1} \varsigma ^{1- \eta } x(\varsigma ) d\varsigma \end{aligned}$$

### Definition 2

^23,24^ Assume that *x*(*t*) is a function that is not constantly differentiable. For a exponential decay kernel in the Riemann–Liouville sense is given as;

3 $$\begin{aligned} _0^{FFE}D_t^{\alpha ,\eta } x(t) = \frac{H(\alpha )}{\Gamma (1- \alpha )} \frac{d}{dt^\eta } \int _0^t \exp \left[ -\frac{\alpha }{1-\alpha }(t-\varsigma )^{- \alpha }\right] x(\varsigma ) d\varsigma \end{aligned}$$

where $\alpha > 0, \eta \le 1$, and $H(0) = 1 = H(1)$. The corresponding exponential decay kernel fractal-fractional integral of order $(\alpha ,\eta )$ is given as

4 $$\begin{aligned} _0^{FFE}I^{\alpha , \eta } x(t) = \frac{\eta (1-\alpha )t^{\eta - 1}x(t)}{H(\alpha )} + \frac{\alpha \eta }{H(\alpha )}\int _0^t \varsigma ^{\alpha -1}x(\varsigma ) d\varsigma \end{aligned}$$

### Definition 3

^23,24^ Assume that *x*(*t*) is a function that is not constantly differentiable. For a Mittag-Leffler kernel in the Riemann–Liouville sense is given as;

5 $$\begin{aligned} _0^{FFM}D_t^{\alpha ,\eta } x(t) = \frac{AB(\alpha )}{(1- \alpha )} \frac{d}{dt^\eta } \int _0^t E_{\alpha } \left[ -\frac{\alpha }{1-\alpha }(t-\varsigma )^{\alpha }\right] x(\varsigma ) d\varsigma \end{aligned}$$

where $0 < \alpha , \eta \le 1$, $E_{\alpha }$ is the Mittag-Leffler function and $AB(\alpha ) = 1 - \alpha + \frac{\alpha }{\Gamma (\alpha )}$ is a normalization function.

The corresponding Mittag-Leffler kernel fractal-fractional integral of order $(\alpha ,\eta )$ is given as

6 $$\begin{aligned} _0^{FFM}I^{\alpha , \eta } x(t) = \frac{\eta (1-\alpha )}{AB(\alpha )}t^{1-\eta } x(t) + \frac{\eta \alpha }{AB(\alpha )\Gamma (\alpha )} \int _0^t (t- \varsigma )^{\alpha - 1} \varsigma ^{1- \eta } x(\varsigma ) d \varsigma \end{aligned}$$

## Diabetes model with fractional derivative

For our motivation, we consider the glucose-insulin-glucagon system given in^8^, which explains the relation and role of insulin and glucagon in maintaining the glucose level in the human body to overcome the risk of death. We construct the fractional order model in followings equations

7 $$\begin{aligned} {}_0^{FFM}D_t^{\psi ,\eta } G= & {} \omega - bG - \frac{\delta I G}{\alpha G + 1} + \delta _i J \nonumber \\ {}_0^{FFM}D_t^{\psi ,\eta } I= & {} \frac{\gamma \beta G^2}{e + G^2} - \mu I \nonumber \\ {}_0^{FFM}D_t^{\psi ,\eta } \beta= & {} \rho \beta \left( 1- \frac{\beta }{v}\right) \nonumber \\ {}_0^{FFM}D_t^{\psi ,\eta } \alpha= & {} \rho _i \alpha \left( 1- \frac{\alpha }{v_i}\right) \nonumber \\ {}_0^{FFM}D_t^{\psi ,\eta } J= & {} - \gamma _i \alpha (G - G_t) - \mu _i J \end{aligned}$$

with initial condition $G(0) = G_0 \ge 0, I(0) = I_0 \ge 0, \beta (0) = \beta _0 \ge 0, \alpha (0) = \alpha _0 \ge 0, J(0) = J_0 \ge 0$ Assume that glucagon is produced by the $\alpha$-cells at low glucose concentrations in order to increase hepatic glucose synthesis, which raises blood glucose levels. An excessive rise in blood glucose levels is prevented by the production of insulin by the $\beta$-cells. *G*(*t*) represents the dynamics of glucose. The term $\delta _i J$ refers to glucagon’s effect on liver gluconeogenesis, which produces glucose. The blood glucose level increases at a rate $\omega$ (the rate of glucose generation by the liver and kidneys) and falls at a rate *bG* (independent of insulin) and $\delta$ (the rate of glucose uptake as a result of insulin sensitivity). where $\gamma$ is the maximum rate of insulin secreted by $\beta$-cells and $\mu I$ is the rate of kidney clearance of insulin. The dynamics of insulin are represented by *I*(*t*). We assumed a logistic equation, where $\rho$ and $\rho _i$ represent the growth rates of the $\beta$ and the $\alpha$ cell masses, respectively. *v* and $v_i$ represent the carrying capacities of the $\beta$ and the $\alpha$ cell masses, respectively. The glucagon *J*(*t*) is released if the glucose level falls below a specific threshold $(G < G_l)$.

## Analysis of proposed model

### Positivity and boundedness of solutions

Here, we demonstrate the suggested model’s positivity and boundedness.

#### Theorem 1

Assume the initial condition be

8 $$\begin{aligned} \{G(0),I(0),\beta (0),\alpha (0),J(0)\} \subset \varpi , \end{aligned}$$

then if the solutions $\{G,I,\beta ,\alpha ,J\}$ exist, they are all positive for all $t \ge 0$.

#### Proof

Start with the basic analysis to demonstrate that responses are superior because they demonstrate real-world issues with positive values using the methodology described in^25,26^. This section looks at the conditions necessary for the proposed model to provide positive results. We’ll describe the norm

9 $$\begin{aligned} \Vert h \Vert _{\infty } = \sup _{t \in D_h} \vert h(t) \vert \end{aligned}$$

where the domain of *h* is $D_h$. Let’s begin with the *G*(*t*) class

10 $$\begin{aligned} {}_0^{FFM}D_t^{\psi ,\eta } G= & {} \omega - bG - \frac{\delta I G}{\alpha G + 1} + \delta _i J, \forall t \ge 0 \nonumber \\\ge & {} -\left( b + \frac{\delta \vert I \vert }{\alpha G + 1} \right) G, \forall t \ge 0 \nonumber \\\ge & {} -\left( b + \frac{\delta \sup _{t \in D_I} \vert I \vert }{\alpha G + 1} \right) G, \forall t \ge 0 \nonumber \\\ge & {} -\left( b + \frac{\delta \Vert I \Vert _{\infty }}{\alpha G + 1} \right) G, \forall t \ge 0 \end{aligned}$$

This yield

11 $$\begin{aligned} G(t)\ge & {} G(0) E_{\psi } \left( - \frac{r^{1-\eta } \psi \left[ b + \frac{\delta \Vert I \Vert _{\infty }}{\alpha G + 1} \right] t^{\psi }}{AB(\psi ) - (1- \psi )\left[ b + \frac{\delta \Vert I \Vert _{\infty }}{\alpha G + 1} \right] }\right) , \forall t \ge 0 \end{aligned}$$

where the time component is *r*. This illustrates that for any $t \ge 0$, *G*(*t*) is positive. For the function *I*(*t*)

12 $$\begin{aligned} _0^{FFM}D_t^{\psi ,\eta } I= & {} \frac{\gamma \beta G^2}{e + G^2} - \mu I, \forall t \ge 0 \nonumber \\\ge & {} - (\mu ) I, \forall t \ge 0 \end{aligned}$$

This yield

13 $$\begin{aligned} I(t)\ge & {} I(0) E_{\psi } \left( - \frac{r^{1-\eta } \psi \left[ \mu \right] t^{\psi }}{AB(\psi ) - (1- \psi )\left[ \mu \right] }\right) , \forall t \ge 0 \end{aligned}$$

where the time component is *r*. This illustrates that for any $t \ge 0$, *I*(*t*) is positive. For the function $\beta (t)$

14 $$\begin{aligned} {}_0^{FFM}D_t^{\psi ,\eta } \beta= & {} \rho \beta \left( 1- \frac{\beta }{v}\right) , \forall t \ge 0 \nonumber \\\ge & {} - \left[ \rho \left( \frac{\vert \beta \vert }{v} - 1\right) \right] \beta , \forall t \ge 0 \nonumber \\\ge & {} - \left[ \rho \left( \frac{\sup _{t \in D_{\beta }} \vert \beta \vert }{v} - 1\right) \right] \beta , \forall t \ge 0 \nonumber \\\ge & {} - \left[ \rho \left( \frac{\Vert \beta \Vert _{\infty }}{v} - 1\right) \right] \beta , \forall t \ge 0 \end{aligned}$$

This yield

15 $$\begin{aligned} \beta (t)\ge & {} \beta (0) E_{\psi } \left( - \frac{r^{1-\eta } \psi \left[ \rho \left( \frac{\Vert \beta \Vert _{\infty }}{v} - 1\right) \right] t^{\psi }}{AB(\psi ) - (1- \psi )\left[ \rho \left( \frac{\Vert \beta \Vert _{\infty }}{v} - 1\right) \right] }\right) , \forall t \ge 0 \end{aligned}$$

where the time component is *r*. This illustrates that for any $t \ge 0$, $\beta (t)$ is positive. For the function $\alpha (t)$

16 $$\begin{aligned} _0^{FFM}D_t^{\psi ,\eta } \alpha= & {} \rho _i \alpha \left( 1- \frac{\alpha }{v_i}\right) , \forall t \ge 0 \nonumber \\\ge & {} - \left[ \rho _i \left( \frac{\vert \alpha \vert }{v_i} - 1\right) \right] \alpha , \forall t \ge 0 \nonumber \\\ge & {} - \left[ \rho _i \left( \frac{\sup _{t \in D_{\alpha }} \vert \alpha \vert }{v_i} - 1\right) \right] \alpha , \forall t \ge 0 \nonumber \\\ge & {} - \left[ \rho _i \left( \frac{\Vert \alpha \Vert _{\infty }}{v_i} - 1\right) \right] \alpha , \forall t \ge 0 \end{aligned}$$

This yield

17 $$\begin{aligned} \alpha (t)\ge & {} \alpha (0) E_{\psi } \left( - \frac{r^{1-\eta } \psi \left[ \rho _i \left( \frac{\Vert \alpha \Vert _{\infty }}{v_i} - 1 \right) \right] t^{\psi }}{AB(\psi ) - (1- \psi )\left[ \rho _i \left( \frac{\Vert \alpha \Vert _{\infty }}{v_i} - 1 \right) \right] }\right) , \forall t \ge 0 \end{aligned}$$

where the time component is *r*. This illustrates that for any $t \ge 0$, $\alpha (t)$ is positive. For the function *J*(*t*)

18 $$\begin{aligned} _0^{FFM}D_t^{\psi ,\eta } J= & {} - \gamma _i \alpha (G - G_t) - \mu _i J, \forall t \ge 0 \nonumber \\\ge & {} - (\mu _i) J, \forall t \ge 0 \end{aligned}$$

This yield

19 $$\begin{aligned} I(t)\ge & {} I(0) E_{\psi } \left( - \frac{r^{1-\eta } \psi \left[ \mu \right] t^{\psi }}{AB(\psi ) - (1- \psi )\left[ \mu \right] }\right) , \forall t \ge 0 \end{aligned}$$

where the time component is *r*. This illustrates that for any $t \ge 0$, *J*(*t*) is positive. $\square$

### Positive invariant regions

#### Theorem 2

The diabetes fractional order model have distinct solution and constrained in $R_+^5$.

#### Proof

System given in (7) is investigated with positive solution given as follows:

20 $$\begin{aligned} _0^{FFM}D_t^{\psi ,\eta } G(t)|_{G=0}= & {} \omega + \delta _i J \ge 0, \end{aligned}$$

21 $$\begin{aligned} _0^{FFM}D_t^{\psi ,\eta } I(t)|_{I=0}= & {} \frac{\gamma \beta G^2}{e + G^2} \ge 0, \end{aligned}$$

22 $$\begin{aligned} _0^{FFM}D_t^{\psi ,\eta } \beta (t)|_{\beta =0}= & {} 0, \end{aligned}$$

23 $$\begin{aligned} _0^{FFM}D_t^{\psi ,\eta } \alpha (t)|_{\alpha =0}= & {} 0, \end{aligned}$$

24 $$\begin{aligned} _0^{FFM}D_t^{\psi ,\eta } J(t)|_{J=0}= & {} \gamma _i \alpha (G_t - G) \ge 0. \end{aligned}$$

If $(G(0),I(0),\beta (0),\alpha (0),J(0)) \in R_+^5$, so that the solution must be from hyperplane. The domain $R_+^5$ is a positive invariant with non-negative orthant because the vector field is enclosed with each hyperplane. $\square$

### Existence and uniqueness analysis

The most crucial application of non-linear functional analysis is the use of fixed point theorems to demonstrate the existence of any non-linear system. Using fixed point contractions, non-linear functional analysis shows the point at which every given non-linear system exists. Fixed point mappings that are defined in Banach space ensure thorough investigation of the existence of unique solutions. The examined model (7) has at least one solution in $[0,{\mathbb {T}}]$ according to a fixed point mapping theorem^27^. Consider the system (7) as

25 $$\begin{aligned} {}_0^{FFM}D_t^{\psi ,\eta } G= & {} \omega - bG - \frac{\delta I G}{\alpha G + 1} + \delta _i J = {\bar{G}}(t,G(t)), \nonumber \\ {}_0^{FFM}D_t^{\psi ,\eta } I= & {} \frac{\gamma \beta G^2}{e + G^2} - \mu I = {\bar{I}}(t,I(t)), \nonumber \\ {}_0^{FFM}D_t^{\psi ,\eta } \beta= & {} \rho \beta \left( 1- \frac{\beta }{v}\right) = {\bar{\beta }}(t,\beta (t)), \nonumber \\ {}_0^{FFM}D_t^{\psi ,\eta } \alpha= & {} \rho _i \alpha \left( 1- \frac{\alpha }{v_i}\right) = {\bar{\alpha }}(t,\alpha (t)), \nonumber \\ {}_0^{FFM}D_t^{\psi ,\eta } J= & {} - \gamma _i \alpha (G - G_t) - \mu _i J = {\bar{J}}(t,J(t)). \end{aligned}$$

The following is a reformulation of (25) in the form of a Fractal-Fractional integral for the Mittag-Leffler kernel as expressed in (6).

26 $$\begin{aligned} G(t)= & {} G(0) + \frac{\eta (1-\psi )t^{1-\eta }}{AB(\psi )} {\bar{G}}(t,G(t)) + \frac{\eta \psi }{AB(\psi )\Gamma (\psi )} \int _0^t (t- \varsigma )^{\psi - 1} \varsigma ^{1- \eta } {\bar{G}}(\varsigma , G(\varsigma )) d \varsigma = A_1 + A_2,\nonumber \\ I(t)= & {} I(0) + \frac{\eta (1-\psi )t^{1-\eta }}{AB(\psi )} {\bar{I}}(t,I(t)) + \frac{\eta \psi }{AB(\psi )\Gamma (\psi )} \int _0^t (t- \varsigma )^{\psi - 1} \varsigma ^{1- \eta } {\bar{I}}(\varsigma , I(\varsigma )) d \varsigma = B_1 + B_2,\nonumber \\ \beta (t)= & {} \beta (0) + \frac{\eta (1-\psi )t^{1-\eta }}{AB(\psi )} {\bar{\beta }}(t,\beta (t)) + \frac{\eta \psi }{AB(\psi )\Gamma (\psi )} \int _0^t (t- \varsigma )^{\psi - 1} \varsigma ^{1- \eta } {\bar{\beta }}(\varsigma , \beta (\varsigma )) d \varsigma = C_1 + C_2,\nonumber \\ \alpha (t)= & {} \alpha (0) + \frac{\eta (1-\psi )t^{1-\eta }}{AB(\psi )} {\bar{\alpha }}(t,\alpha (t)) + \frac{\eta \psi }{AB(\psi )\Gamma (\psi )} \int _0^t (t- \varsigma )^{\psi - 1} \varsigma ^{1- \eta } {\bar{\alpha }}(\varsigma , \alpha (\varsigma )) d \varsigma = D_1 + D_2,\nonumber \\ J(t)= & {} J(0) + \frac{\eta (1-\psi )t^{1-\eta }}{AB(\psi )} {\bar{J}}(t,J(t)) + \frac{\eta \psi }{AB(\psi )\Gamma (\psi )} \int _0^t (t- \varsigma )^{\psi - 1} \varsigma ^{1- \eta } {\bar{J}}(\varsigma , J(\varsigma )) d \varsigma = E_1 + E_2, \end{aligned}$$

where

27 $$\begin{aligned} A_1= & {} G(0) + \frac{\eta (1-\psi )t^{1-\eta }}{AB(\psi )} {\bar{G}}(t,G(t)), \hspace{0.5cm} A_2 = \frac{\eta \psi }{AB(\psi )\Gamma (\psi )} \int _0^t (t- \varsigma )^{\psi - 1} \varsigma ^{1- \eta } {\bar{G}}(\varsigma , G(\varsigma )) d \varsigma \nonumber \\ B_1= & {} I(0) + \frac{\eta (1-\psi )t^{1-\eta }}{AB(\psi )} {\bar{I}}(t,I(t)), \hspace{0.5cm} B_2 = \frac{\eta \psi }{AB(\psi )\Gamma (\psi )} \int _0^t (t- \varsigma )^{\psi - 1} \varsigma ^{1- \eta } {\bar{I}}(\varsigma , I(\varsigma )) d \varsigma \nonumber \\ C_1= & {} \beta (0) + \frac{\eta (1-\psi )t^{1-\eta }}{AB(\psi )} {\bar{\beta }}(t,\beta (t)), \hspace{0.5cm} C_2 = \frac{\eta \psi }{AB(\psi )\Gamma (\psi )} \int _0^t (t- \varsigma )^{\psi - 1} \varsigma ^{1- \eta } {\bar{\beta }}(\varsigma , \beta (\varsigma )) d \varsigma \nonumber \\ D_1= & {} \alpha (0) + \frac{\eta (1-\psi )t^{1-\eta }}{AB(\psi )} {\bar{\alpha }}(t,\alpha (t)), \hspace{0.5cm} D_2 = \frac{\eta \psi }{AB(\psi )\Gamma (\psi )} \int _0^t (t- \varsigma )^{\psi - 1} \varsigma ^{1- \eta } {\bar{\alpha }}(\varsigma , \alpha (\varsigma )) d \varsigma \nonumber \\ E_1= & {} J(0) + \frac{\eta (1-\psi )t^{1-\eta }}{AB(\psi )} {\bar{J}}(t,J(t)), \hspace{0.5cm} E_2 = \frac{\eta \psi }{AB(\psi )\Gamma (\psi )} \int _0^t (t- \varsigma )^{\psi - 1} \varsigma ^{1- \eta } {\bar{J}}(\varsigma , J(\varsigma )) d \varsigma \end{aligned}$$

We prove the primary component of governing Eq. (26), $M(A_1,B_1,C_1,D_1,E_1)$ as contraction maps and $N(A_2,B_2,C_2,D_2,E_2)$ as continuous compact integral parts using Krasnoselski’s fixed point theorem.

#### Theorem 3

The non-linear map $M(A_1,B_1,C_1,D_1,E_1):[0,{\mathbb {T}}] \times {\mathbb {R}} \times {\mathbb {R}} \rightarrow {\mathbb {R}}^5$ given in (27) ensures Lipschitz contractive condition for constants $P_A,P_B,P_C,P_D,P_E > 0$.

#### Proof

Consider the operator $M(A_1,B_1,C_1,D_1,E_1):[0,{\mathbb {T}}] \times {\mathbb {R}} \times {\mathbb {R}} \rightarrow {\mathbb {R}}^5$ defined on a fully normed space. Where the norm is

28 $$\begin{aligned} \Vert (G,I,\beta , \alpha , J) \Vert = \max _{t \in [0, {\mathbb {T}}]} \Vert G(t) + I(t) + \beta (t) + \alpha (t) + J(t) \Vert , \hspace{0.5cm} G,I,\beta ,\alpha ,J \in [0,{\mathbb {T}}] \end{aligned}$$

(i) Firstly, we will show that $M(A_1,B_1,C_1,D_1,E_1)$ is a contraction map. For *G*(*t*) and ${\hat{G}}(t)$, we have
  29 $$\begin{aligned} \Vert A(G,I,\beta ,\alpha ,J)(t) - A({\hat{G}},I,\beta ,\alpha ,J)(t) \Vert= & {} \Vert \{ \omega - bG - \frac{\delta I G}{\alpha G + 1} + \delta _i J \} - \{ \omega - b {\hat{G}}- \frac{\delta I {\hat{G}}}{\alpha G + 1} + \delta _i J \} \Vert \nonumber \\= & {} \Vert -b(G-{\hat{G}}) - \frac{\delta I}{\alpha G + 1}(G - {\hat{G}}) \Vert \nonumber \\\le & {} \Vert b + \frac{\delta I}{\alpha G + 1} \Vert \Vert (G - {\hat{G}}) \Vert \nonumber \\\le & {} P_A \Vert (G - {\hat{G}}) \Vert \end{aligned}$$
  where $P_A = \Vert b + \frac{\delta I}{\alpha G + 1} \Vert$. Using this approach, we have
  $$\begin{aligned} {\left\{ \begin{array}{ll} \Vert B(G,I,\beta ,\alpha ,J)(t) - B(G,{\hat{I}},\beta ,\alpha ,J)(t) \Vert \le P_B \Vert (I - {\hat{I}}) \Vert \\ \Vert C(G,I,\beta ,\alpha ,J)(t) - C(G,I,{\hat{\beta }},\alpha ,J)(t) \Vert \le P_C \Vert (\beta - {\hat{\beta }}) \Vert \\ \Vert D(G,I,\beta ,\alpha ,J)(t) - D(G,I,\beta ,{\hat{\alpha }},J)(t) \Vert \le P_D \Vert (\alpha - {\hat{\alpha }}) \Vert \\ \Vert E(G,I,\beta ,\alpha ,J)(t) - E(G,I,\beta ,\alpha ,{\hat{J}})(t) \Vert \le P_E \Vert (J - {\hat{J}}) \Vert \\ \end{array}\right. } \end{aligned}$$
  where $P_B = \Vert \mu \Vert , P_C = \Vert \rho \left( 1- \frac{\beta }{v}\right) \Vert , P_D = \Vert \rho _i \left( 1- \frac{\alpha }{v_i}\right) \Vert , P_E = \Vert \mu _i \Vert$ This implies that, for the operator $M(G,I,\beta ,\alpha ,J)$, we have
  30 $$\begin{aligned} \Vert M(G,I,\beta ,\alpha ,J) - M({\hat{G}},{\hat{I}},{\hat{\beta }},{\hat{\alpha }},{\hat{J}}) \Vert= & {} \frac{\eta (1-\psi )t^{\eta -1}}{AB(\psi )}\max _{t \in [0,{\mathbb {T}}]} \vert (G,I,\beta ,\alpha ,J)(t) - ({\hat{G}},{\hat{I}},{\hat{\beta }},{\hat{\alpha }},{\hat{J}})(t) \vert \nonumber \\\le & {} \frac{\eta (1-\psi )t^{\eta -1}}{AB(\psi )} \Vert (G,I,\beta ,\alpha ,J)(t) - ({\hat{G}},{\hat{I}},{\hat{\beta }},{\hat{\alpha }},{\hat{J}})(t) \Vert \nonumber \\\le & {} \frac{\eta (1-\psi )t^{\eta -1}}{AB(\psi )}P \end{aligned}$$
  where $P = \max [P_A,P_B,P_C,P_D,P_E] < 1$ is a Lipschitz constant. This implies *M*(*A*, *B*, *C*, *D*, *E*) is a non-expansive operator.
(ii) Now we will show that $N(A_2,B_2,C_2,D_2,E_2)$ is continuously compact. The absolute modulus of all positively bounded continuous operators *A*, *B*, *C*, *D*, *E* specified in (27) given by the non-zero positive constants $\wp _A, \wp _B, \wp _C, \wp _D, \wp _E, \aleph _A, \aleph _B, \aleph _C, \aleph _D, \aleph _E$ meeting the following bounded-ness inequalities, illustrates the compactness of the operator $N(A_2,B_2,C_2,D_2,E_2)$.
  31 $$\begin{aligned} \vert A(t,G) \vert \le \wp _A \Vert G \Vert + \aleph _A\nonumber \\ \vert B(t,I) \vert \le \wp _B \Vert I \Vert + \aleph _B\nonumber \\ \vert C(t,\beta ) \vert \le \wp _C \Vert \beta \Vert + \aleph _C\nonumber \\ \vert D(t,\alpha ) \vert \le \wp _D \Vert \alpha \Vert + \aleph _D\nonumber \\ \vert E(t,J) \vert \le \wp _E \Vert J \Vert + \aleph _E \end{aligned}$$
  Suppose that $\chi$ is a closed subset of ${\mathbb {Z}}$ as
  32 $$\begin{aligned} \chi = \{(A,B,C,D,E) \in {\mathbb {Z}} / \Vert A,B,C,D,E \vert \le \Lambda , \Lambda > 0 \} \end{aligned}$$
  For $(A,B,C,D,E) \in \chi$, we find
  33 $$\begin{aligned} \Vert A_2(t,G) \Vert= & {} \max _{t \in [0,{\mathbb {T}}]} \vert \frac{\psi \eta }{AB(\psi )\Gamma (\psi )} \int _0^t (t- \varsigma )^{\psi - 1} \varsigma ^{1 - \eta } A(\varsigma , G(\varsigma )) d \varsigma \vert \nonumber \\\le & {} \frac{\tau ^{\psi ,\eta }}{AB(\psi )\Gamma (\psi )} \int _0^\tau (\tau - \varsigma )^{\psi - 1} \varsigma ^{1 - \eta } A(\varsigma , G(\varsigma )) d \varsigma \vert \nonumber \\\le & {} \frac{\tau ^{\psi ,\eta }}{AB(\psi )\Gamma (\psi )} \wp _A \Lambda + \aleph _A \end{aligned}$$
  Similarly, we find
  $$\begin{aligned} {\left\{ \begin{array}{ll} \Vert B_2(t,I) \Vert \le \frac{\tau ^{\psi ,\eta }}{AB(\psi )\Gamma (\psi )} \wp _B \Lambda + \aleph _B \\ \Vert C_2(t,\beta ) \Vert \le \frac{\tau ^{\psi ,\eta }}{AB(\psi )\Gamma (\psi )} \wp _C \Lambda + \aleph _C \\ \Vert D_2(t,\alpha ) \Vert \le \frac{\tau ^{\psi ,\eta }}{AB(\psi )\Gamma (\psi )} \wp _D \Lambda + \aleph _D \\ \Vert E_2(t,J) \Vert \le \frac{\tau ^{\psi ,\eta }}{AB(\psi )\Gamma (\psi )} \wp _E \Lambda + \aleph _E \\ \end{array}\right. } \end{aligned}$$
  proceeding this process, we find the maximum norm of $\Vert \Xi (A_2,B_2,C_2,D_2,E_2) \Vert$ as,
  34 $$\begin{aligned} \Vert \Xi (A_2,B_2,C_2,D_2,E_2) \Vert \le \{ [\wp _A + \wp _B + \wp _C + \wp _D + \wp _E]\Lambda + \aleph _A + \aleph _B + \aleph _C + \aleph _D + \aleph _E \} = \xi \end{aligned}$$
  where $\xi$ is a positive constant. Therefore,
  35 $$\begin{aligned} \Vert \Xi (A_2,B_2,C_2,D_2,E_2) \Vert \le \xi \Rightarrow \Xi \end{aligned}$$
  is a uniformly bounded operator. Now we will prove that $\Xi$ is equi-continuous for $t_x < t_y \in [0, {\mathbb {T}}]$. For this purpose, we have for $t_x < t_y \in [0, {\mathbb {T}}]$
  36 $$\begin{aligned} \vert A_2(t_2,G) - A_2(t_1,G) \vert= & {} \frac{\psi \eta }{AB(\psi )\Gamma (\psi )} \vert \int _0^{t_y} (t- \varsigma )^{\psi - 1} \varsigma ^{1 - \eta } A(\varsigma , G(\varsigma )) d \varsigma \nonumber \\- & {} \int _0^{t_x} (t- \varsigma )^{\psi - 1} \varsigma ^{1 - \eta } A(\varsigma , G(\varsigma )) d \varsigma \vert \nonumber \\\le & {} \frac{\psi \eta }{AB(\psi )\Gamma (\psi )} \left[ \int _0^{t_y} (t- \varsigma )^{\psi - 1} \varsigma ^{1 - \eta } d \varsigma - \int _0^{t_x} (t- \varsigma )^{\psi - 1} \varsigma ^{1 - \eta } d \varsigma \right] (\wp _A \Lambda + \aleph _A) \nonumber \\\le & {} \frac{\wp _A \Lambda + \aleph _A}{AB(\psi )\Gamma (\psi )} \left[ t_2^{\psi ,\eta } - t_1^{\psi ,\eta } \right] \end{aligned}$$
  Similarly,
  $$\begin{aligned} {\left\{ \begin{array}{ll} \vert B_2(t_2,I) - B_2(t_1,I) \vert \le \frac{\wp _B \Lambda + \aleph _B}{AB(\psi )\Gamma (\psi )} \left[ t_2^{\psi ,\eta } - t_1^{\psi ,\eta } \right] ,\\ \vert C_2(t_2,\beta ) - C_2(t_1,\beta ) \vert \le \frac{\wp _C \Lambda + \aleph _C}{AB(\psi )\Gamma (\psi )} \left[ t_2^{\psi ,\eta } - t_1^{\psi ,\eta } \right] ,\\ \vert D_2(t_2,\alpha ) - D_2(t_1,\alpha ) \vert \le \frac{\wp _D \Lambda + \aleph _D}{AB(\psi )\Gamma (\psi )} \left[ t_2^{\psi ,\eta } - t_1^{\psi ,\eta } \right] ,\\ \vert E_2(t_2,J) - E_2(t_1,J) \vert \le \frac{\wp _E \Lambda + \aleph _E}{AB(\psi )\Gamma (\psi )} \left[ t_2^{\psi ,\eta } - t_1^{\psi ,\eta } \right] . \end{array}\right. } \end{aligned}$$
  Since $t_2 \rightarrow t_1$ is independent of $(G,I,\beta ,\alpha ,J)$. This implies that
  37 $$\begin{aligned} \Vert \Xi (A_2,B_2,C_2,D_2,E_2)(t_2) - \Xi (A_2,B_2,C_2,D_2,E_2)(t_1) \Vert \rightarrow 0 \end{aligned}$$
  $\Rightarrow \Xi (A_2,B_2,C_2,D_2,E_2)$ is a completely continuous, equi-continuous operator. $\Rightarrow \Xi (A_2,B_2,C_2,D_2,E_2)$ is relatively compact by Arzela’s theorem. As a result, the Krasnoselski theorem follows, which states that the contraction and continuity of the operators *M* and *N* ensure the existence of a single unique solution.

$$\square $$

#### Theorem 4

The model (7) has a unique solution if

38 $$\begin{aligned} \frac{\tau ^{\psi ,\eta }}{AB(\psi )\Gamma (\psi )} P \le 1 \end{aligned}$$

where $$P = \max \{P_A,P_B,P_C,P_D,P_E \}$$.

#### Proof

Establish an operator $H = (H_1,H_2,H_3,H_4,H_5):{\mathbb {Z}} \rightarrow {\mathbb {Z}}$ utilizing (31) as:

39 $$\begin{aligned} H_1(G,I,\beta ,\alpha ,J)(t)= & {} G(0) + \frac{\eta (1-\psi )t^{1-\eta }}{AB(\psi )} A(t,G(t)) + \frac{\eta \psi }{AB(\psi )\Gamma (\psi )} \int _0^t (t- \varsigma )^{\psi - 1} \varsigma ^{1- \eta } A(\varsigma , G(\varsigma )) d \varsigma ,\nonumber \\ H_2(G,I,\beta ,\alpha ,J)(t)= & {} I(0) + \frac{\eta (1-\psi )t^{1-\eta }}{AB(\psi )} B(t,I(t)) + \frac{\eta \psi }{AB(\psi )\Gamma (\psi )} \int _0^t (t- \varsigma )^{\psi - 1} \varsigma ^{1- \eta } B (\varsigma , I(\varsigma )) d \varsigma ,\nonumber \\ H_3(G,I,\beta ,\alpha ,J)(t)= & {} \beta (0) + \frac{\eta (1-\psi )t^{1-\eta }}{AB(\psi )} C(t,\beta (t)) + \frac{\eta \psi }{AB(\psi )\Gamma (\psi )} \int _0^t (t- \varsigma )^{\psi - 1} \varsigma ^{1- \eta } C(\varsigma , \beta (\varsigma )) d \varsigma ,\nonumber \\ H_4(G,I,\beta ,\alpha ,J)(t)= & {} \alpha (0) + \frac{\eta (1-\psi )t^{1-\eta }}{AB(\psi )} D(t,\alpha (t)) + \frac{\eta \psi }{AB(\psi )\Gamma (\psi )} \int _0^t (t- \varsigma )^{\psi - 1} \varsigma ^{1- \eta } D(\varsigma , \alpha (\varsigma )) d \varsigma ,\nonumber \\ H_5(G,I,\beta ,\alpha ,J)(t)= & {} J(0) + \frac{\eta (1-\psi )t^{1-\eta }}{AB(\psi )} E(t,J(t)) + \frac{\eta \psi }{AB(\psi )\Gamma (\psi )} \int _0^t (t- \varsigma )^{\psi - 1} \varsigma ^{1- \eta } E(\varsigma , J(\varsigma )) d \varsigma . \end{aligned}$$

For $(G,I,\beta ,\alpha ,J) , ({\bar{G}}, {\bar{I}}, {\bar{\beta }}, {\bar{\alpha }}, {\bar{J}}) \in {\mathbb {Z}}$, and utilizing (39) we have,

40 $$\begin{aligned} \Vert H_1(G,I,\beta ,\alpha ,J)(t) - H_1({\bar{G}}, {\bar{I}}, {\bar{\beta }}, {\bar{\alpha }}, {\bar{J}})(t) \Vert= & {} \frac{\eta (1-\psi )t^{1-\eta }}{AB(\psi )} \Vert A(t,G(t)) - A(t,{\bar{G}}(t))\Vert \nonumber \\+ & {} \frac{\eta \psi }{AB(\psi )\Gamma (\psi )} \int _0^t (t- \varsigma )^{\psi - 1} \varsigma ^{1- \eta } \Vert A(\varsigma , G(\varsigma )) - A(\varsigma , {\bar{G}}(\varsigma )) \Vert d \varsigma ,\nonumber \\\le & {} \frac{\eta (1-\psi )t^{1-\eta }}{AB(\psi )} P_A \Vert G - {\bar{G}} \Vert + \frac{\varsigma ^{\eta , \psi }}{AB(\psi )\Gamma (\psi )} P_A \Vert G - {\bar{G}} \Vert \nonumber \\\le & {} \left[ \frac{\eta (1-\psi )t^{1-\eta }}{AB(\psi )} + \frac{\varsigma ^{\eta , \psi }}{AB(\psi )\Gamma (\psi )} \right] P_A \Vert G - {\bar{G}} \Vert \end{aligned}$$

$$\Vert G - {\bar{G}} \Vert \rightarrow 0$$ when $$ G \rightarrow {\bar{G}}$$. Hence

41 $$\begin{aligned} \Vert H_1(G,I,\beta ,\alpha ,J)(t) - H_1({\bar{G}}, {\bar{I}}, {\bar{\beta }}, {\bar{\alpha }}, {\bar{J}})(t) \Vert\le & {} \left[ \frac{\eta (1-\psi )t^{1-\eta }}{AB(\psi )} + \frac{\varsigma ^{\eta , \psi }}{AB(\psi )\Gamma (\psi )} \right] P_A \le 1 \end{aligned}$$

with

42 $$\begin{aligned} \Vert H_1(G,I,\beta ,\alpha ,J)(t) - H_1({\bar{G}}, {\bar{I}}, {\bar{\beta }}, {\bar{\alpha }}, {\bar{J}})(t) \Vert \left[ 1 - \left( \frac{\eta (1-\psi )t^{1-\eta }}{AB(\psi )} + \frac{\varsigma ^{\eta , \psi }}{AB(\psi )\Gamma (\psi )} \right) P_A \right] \le 0 \end{aligned}$$

Similarly, we find

43 $$\begin{aligned} \Vert H_2(G,I,\beta ,\alpha ,J)(t) - H_2({\bar{G}}, {\bar{I}}, {\bar{\beta }}, {\bar{\alpha }}, {\bar{J}})(t) \Vert \left[ 1 - \left( \frac{\eta (1-\psi )t^{1-\eta }}{AB(\psi )} + \frac{\varsigma ^{\eta , \psi }}{AB(\psi )\Gamma (\psi )} \right) P_B \right]\le & {} 0\nonumber \\ \Vert H_3(G,I,\beta ,\alpha ,J)(t) - H_3({\bar{G}}, {\bar{I}}, {\bar{\beta }}, {\bar{\alpha }}, {\bar{J}})(t) \Vert \left[ 1 - \left( \frac{\eta (1-\psi )t^{1-\eta }}{AB(\psi )} + \frac{\varsigma ^{\eta , \psi }}{AB(\psi )\Gamma (\psi )} \right) P_C \right]\le & {} 0\nonumber \\ \Vert H_4(G,I,\beta ,\alpha ,J)(t) - H_4({\bar{G}}, {\bar{I}}, {\bar{\beta }}, {\bar{\alpha }}, {\bar{J}})(t) \Vert \left[ 1 - \left( \frac{\eta (1-\psi )t^{1-\eta }}{AB(\psi )} + \frac{\varsigma ^{\eta , \psi }}{AB(\psi )\Gamma (\psi )} \right) P_D \right]\le & {} 0\nonumber \\ \Vert H_5(G,I,\beta ,\alpha ,J)(t) - H_5({\bar{G}}, {\bar{I}}, {\bar{\beta }}, {\bar{\alpha }}, {\bar{J}})(t) \Vert \left[ 1 - \left( \frac{\eta (1-\psi )t^{1-\eta }}{AB(\psi )} + \frac{\varsigma ^{\eta , \psi }}{AB(\psi )\Gamma (\psi )} \right) P_E \right]\le & {} 0 \end{aligned}$$

Therefore,

$$\begin{aligned} \Vert H(G,I,\beta ,\alpha ,J)(t) - H({\bar{G}}, {\bar{I}}, {\bar{\beta }}, {\bar{\alpha }}, {\bar{J}})(t) \Vert \le \left[ \frac{\eta (1-\psi )t^{1-\eta }}{AB(\psi )} + \frac{\varsigma ^{\eta \psi }}{AB(\psi )\Gamma (\psi )}\right] P \Vert (G,I,\beta ,\alpha ,J) - ({\bar{G}}, {\bar{I}}, {\bar{\beta }}, {\bar{\alpha }}, {\bar{J}}) \Vert \end{aligned}$$

The contraction map *H* inherits the features of Schauder’s and Krasnoselski’s theorems and confirms our suggested model’s unique fixed point solution. $\square$

#### Remark 1

The derived unique solution is attractive

- if the zero solution $(G,I,\beta ,\alpha ,J)(t) =0$ such that
  44 $$\begin{aligned} \Vert (G,I,\beta ,\alpha ,J)\Vert \le \varepsilon , \hspace{0.5cm} implies \hspace{0.2cm} that \hspace{0.5cm} \lim _{t \rightarrow \infty } (G,I,\beta ,\alpha ,J)(t) = 0 \end{aligned}$$
- if the trivial solution $\varphi (t) = 0$ such that
  $$\begin{aligned} \Vert Z_0 \Vert \le \varepsilon \Rightarrow \lim _{t \rightarrow \infty } z_0 = 0. \end{aligned}$$

### Equilibrium points analysis

The system given in (7) is solved for equilibria. we have

45 $$\begin{aligned} E_1= & {} \left( \frac{\omega }{b},0,0,0, 0 \right) \end{aligned}$$

46 $$\begin{aligned} E_2= & {} \left( \frac{\mu _i \omega + b v_i \gamma _i G_t}{b \mu _i + \delta _i v_i \gamma _i}, 0, 0, v_i, \frac{-(\omega v_i \gamma _i + \delta _i v_i \gamma _i G_t)}{b \mu _i + \delta _i v_i \gamma _i} \right) \end{aligned}$$

### Stability analysis

Global stability is analyzed for the proposed system as follows.

#### Lemma 1

Let $h \in R^+$ represents the continuous function for which any $t \ge t_0$,

47 $$\begin{aligned} _0^{FFM}D_t^{\psi ,\eta } \left( h(t) - h^* - h^* \log \frac{h(t)}{h^*} \right) \le \left( 1 - \frac{h^*}{h(t)}\right) {_0^{FFM}D_t^{\psi ,\eta }} h(t) \end{aligned}$$

$$h^* \in R^+$$, $$\forall \alpha \in (0,1)$$.

#### First derivative of Lyapunov

Lyapunov function for the endemic, $\{G,I,\beta ,\alpha ,J\}$, $L < 0$ is the endemic equilibrium points $E^*$.

##### Theorem 5

The endemic equilibria $E^*$ for the model are globally asymptotically stable, If the reproductive number $R_0 > 1$.

##### Proof

Suppose that the Volterra-type Lyapunov function as:

48 $$\begin{aligned} M = C_1 (G - G^* - G^* \log \frac{G^*}{G}) + C_2 (I - I^* - I^* \log \frac{I^*}{I}) + C_3 (\beta - \beta ^* - \beta ^* \log \frac{\beta ^*}{\beta })\nonumber \\ + C_4 (\alpha - \alpha ^* - \alpha ^* \log \frac{\alpha ^*}{\alpha }) + C_5 (J - J^* - J^* \log \frac{J^*}{J}) \end{aligned}$$

Where $C_i, i=1,2,3,4,5$ are positive constants will be considered later. Then putting Eq. (48) into main system and using Lemma (3.1).

49 $$\begin{aligned} _0^{FFM}D_t^{\psi ,\eta } M\le & {} C_1 \left( \frac{G - G^*}{G} \right) {_0^{FFM}D_t^{\psi ,\eta }} G + C_2 \left( \frac{I-I^*}{I} \right) {_0^{FFM}D_t^{\psi ,\eta }} I + C_3 \left( \frac{\beta -\beta ^*}{\beta } \right) {_0^{FFM}D_t^{\psi ,\eta }} \beta \nonumber \\+ & {} C_4 \left( \frac{\alpha -\alpha ^*}{\alpha } \right) {_0^{FFM}D_t^{\psi ,\eta }} \alpha + C_5 \left( \frac{J-J^*}{J} \right) {_0^{FFM}D_t^{\psi ,\eta }} J \end{aligned}$$

After the substituting the values of the derivative derivatives, we have.

50 $$\begin{aligned} _0^{FFM}D_t^{\psi ,\eta } M\le & {} C_1 \left( \frac{G - G^*}{G} \right) (\omega - bG - \frac{\delta I G}{\alpha G + 1} + \delta _i J) + C_2 \left( \frac{I-I^*}{I} \right) (\frac{\gamma \beta G^2}{e + G^2} - \mu I) + C_3 \left( \frac{\beta -\beta ^*}{\beta } \right) \nonumber \\\times & {} \left( \rho \beta \left( 1- \frac{\beta }{v}\right) \right) + C_4 \left( \frac{\alpha -\alpha ^*}{\alpha } \right) (\rho _i \alpha \left( 1- \frac{\alpha }{v_i}\right) ) + C_5 \left( \frac{J-J^*}{J} \right) (- \gamma _i \alpha (G - G_t) - \mu _i J) \end{aligned}$$

Replacing $G=G-G^*, I=I-I^*, \beta =\beta -\beta ^*, \alpha =\alpha -\alpha ^*, J=J-J^*$, we can have the following

51 $$\begin{aligned} _0^{FFM}D_t^{\psi ,\eta } M\le & {} C_1 \left( \frac{G - G^*}{G} \right) (\omega - b(G-G^*) - \frac{\delta (I-I^*) (G-G^*)}{(\alpha -\alpha ^*) (G-G^*) + 1} + \delta _i (J-J^*)) + C_2 \left( \frac{I-I^*}{I} \right) \nonumber \\\times & {} \left( \frac{\gamma (\beta -\beta ^*) (G-G^*)^2}{e + (G-G^*)^2} - \mu (I-I^*)\right) + C_3 \left( \frac{\beta -\beta ^*}{\beta } \right) \left( \rho (\beta -\beta ^*) \left( 1- \frac{(\beta -\beta ^*)}{v}\right) \right) \nonumber \\+ & {} C_4 \left( \frac{\alpha -\alpha ^*}{\alpha } \right) (\rho _i (\alpha -\alpha ^*) \left( 1- \frac{(\alpha -\alpha ^*)}{v_i}\right) ) + C_5 \left( \frac{J-J^*}{J} \right) (- \gamma _i \alpha ((G-G^*) - G_t)\nonumber \\- & {} \mu _i (J-J^*)) \end{aligned}$$

Now let$$ C_1 = C_2 = C_3 = C_4 = C_5 = 1$$. We can organize the above as follows

52 $$\begin{aligned} _0^{FFM}D_t^{\psi ,\eta } M\le & {} \omega - \omega \frac{G^*}{G} - b \frac{(G-G^*)^2}{G} - \frac{\delta (I-I^*) (G-G^*)^2}{G((\alpha -\alpha ^*) (G-G^*) + 1)} + \delta _i J - \delta _i J^* - \delta _i \frac{G^*}{G}J + \delta _i \frac{G^*}{G}J^* \nonumber \\+ & {} \frac{\gamma (\beta -\beta ^*) (G-G^*)^2}{e + (G-G^*)^2} - \frac{[\gamma (\beta -\beta ^*) (G-G^*)^2]I^*}{[e + (G-G^*)^2]I} - \mu \frac{(I-I^*)^2}{I} + \rho \frac{(\beta - \beta ^*)^2}{\beta } - \rho \frac{(\beta - \beta ^*)^3}{v \beta }\nonumber \\+ & {} \rho _i \frac{(\alpha - \alpha ^*)^2}{\alpha } - \rho _i \frac{(\alpha - \alpha ^*)^3}{v_i \alpha } - \gamma _i \alpha G + \gamma _i \alpha G^* + \gamma _i \alpha G_t + \gamma _i \alpha ^* G - \gamma _i \alpha ^* G^* - \gamma _i \alpha ^* G_t + \gamma _i \alpha G \frac{J^*}{J} \nonumber \\- & {} \gamma _i \alpha G^* \frac{J^*}{J} - \gamma _i \alpha G_t \frac{J^*}{J} - \gamma _i \alpha ^* G \frac{J^*}{J} + \gamma _i \alpha ^* G^* \frac{J^*}{J} + \gamma _i \alpha ^* G_t \frac{J^*}{J} - \mu _i \frac{(J - J^*)^2}{J} \end{aligned}$$

after simplification, we get

53 $$\begin{aligned} _0^{FFM}D_t^{\psi ,\eta } M\le & {} \Omega - \Sigma \end{aligned}$$

where

54 $$\begin{aligned} \Omega= & {} \omega + \delta _i J + \delta _i \frac{G^*}{G}J^* + \frac{\gamma (\beta -\beta ^*) (G-G^*)^2}{e + (G-G^*)^2} + \rho \frac{(\beta - \beta ^*)^2}{\beta } + \rho _i \frac{(\alpha - \alpha ^*)^2}{\alpha }\nonumber \\+ & {} \gamma _i \alpha G^* + \gamma _i \alpha G_t + \gamma _i \alpha ^* G + \gamma _i \alpha G \frac{J^*}{J} + \gamma _i \alpha ^* G^* \frac{J^*}{J} + \gamma _i \alpha ^* G_t \frac{J^*}{J} \end{aligned}$$

and

55 $$\begin{aligned} \Sigma= & {} \omega \frac{G^*}{G} + b \frac{(G-G^*)^2}{G} + \frac{\delta (I-I^*) (G-G^*)^2}{G((\alpha -\alpha ^*) (G-G^*) + 1)} + \delta _i J^* + \delta _i \frac{G^*}{G}J + \frac{[\gamma (\beta -\beta ^*) (G-G^*)^2]I^*}{[e + (G-G^*)^2]I} \nonumber \\+ & {} \mu \frac{(I-I^*)^2}{I} + \rho \frac{(\beta - \beta ^*)^3}{v \beta } + \rho _i \frac{(\alpha - \alpha ^*)^3}{v_i \alpha } + \gamma _i \alpha G + \gamma _i \alpha ^* G^* + \gamma _i \alpha ^* G_t + \gamma _i \alpha G^* \frac{J^*}{J} + \gamma _i \alpha G_t \frac{J^*}{J} \nonumber \\+ & {} \gamma _i \alpha ^* G \frac{J^*}{J} + \mu _i \frac{(J - J^*)^2}{J} \end{aligned}$$

it is concluded that if $\Omega < \Sigma$ this yields $_0^{FFM}D_t^{\psi ,\eta } M < 0$ however when $G=G^*, I=I^*, \beta =\beta ^*, \alpha =\alpha ^*, J=J^*$ so $\Omega - \Sigma = 0$ , $_0^{FFM}D_t^{\psi ,\eta } M = 0$ $\square$

#### Second derivative of Lyapunov

56 $$\begin{aligned} _0^{FFM}D_t^{\psi ,\eta }[_0^{FFM}D_t^{\psi ,\eta }] M\le & {} \left( \frac{_0^{FFM}D_t^{\psi ,\eta } G}{G} \right) ^2 G^* + \left( \frac{_0^{FFM}D_t^{\psi ,\eta } I}{I} \right) ^2 I^* + \left( \frac{_0^{FFM}D_t^{\psi ,\eta } \beta }{\beta } \right) ^2 \beta ^* \nonumber \\+ & {} \left( \frac{_0^{FFM}D_t^{\psi ,\eta } \alpha }{\alpha } \right) ^2 \alpha ^* + \left( \frac{_0^{FFM}D_t^{\psi ,\eta } J}{J} \right) ^2 J^* + \left( 1 + \frac{G^*}{G} \right) {_0^{FFM}D_t^{\psi ,\eta }} [{_0^{FFM}D_t^{\psi ,\eta }} G] \nonumber \\+ & {} \left( 1 + \frac{I^*}{I} \right) {_0^{FFM}D_t^{\psi ,\eta }} [{_0^{FFM}D_t^{\psi ,\eta }} I] +\left( 1 + \frac{\beta ^*}{\beta } \right) {_0^{FFM}D_t^{\psi ,\eta }} [{_0^{FFM}D_t^{\psi ,\eta }} \beta ] \nonumber \\+ & {} \left( 1 + \frac{\alpha ^*}{\alpha } \right) {_0^{FFM}D_t^{\psi ,\eta }} [{_0^{FFM}D_t^{\psi ,\eta }} \alpha ] + \left( 1 + \frac{J^*}{J} \right) {_0^{FFM}D_t^{\psi ,\eta }} [{_0^{FFM}D_t^{\psi ,\eta }} J] \end{aligned}$$

where

57 $$\begin{aligned} {_0^{FFM}D_t^{\psi ,\eta }} [{_0^{FFM}D_t^{\psi ,\eta }} G]= & {} - b ({_0^{FFM}D_t^{\psi ,\eta }} G) + \delta _i ({_0^{FFM}D_t^{\psi ,\eta }} J) - \frac{\Phi }{(\alpha G + 1)^2}\nonumber \\ {_0^{FFM}D_t^{\psi ,\eta }} [{_0^{FFM}D_t^{\psi ,\eta }} I]= & {} \frac{(e)(\gamma ({_0^{FFM}D_t^{\psi ,\eta }} \beta )G^2 + 2 \gamma \beta G ({_0^{FFM}D_t^{\psi ,\eta }} G)) + \gamma ({_0^{FFM}D_t^{\psi ,\eta }} \beta ) G^4}{(e + G^2)^2} - \mu ({_0^{FFM}D_t^{\psi ,\eta }} I) \nonumber \\ {_0^{FFM}D_t^{\psi ,\eta }} [{_0^{FFM}D_t^{\psi ,\eta }} \beta ]= & {} \rho ({_0^{FFM}D_t^{\psi ,\eta }} \beta ) - \frac{2 \rho \beta ({_0^{FFM}D_t^{\psi ,\eta }} \beta ) }{v}\nonumber \\ {_0^{FFM}D_t^{\psi ,\eta }} [{_0^{FFM}D_t^{\psi ,\eta }} \alpha ]= & {} \rho _i ({_0^{FFM}D_t^{\psi ,\eta }} \alpha ) - \frac{2 \rho _i \alpha ({_0^{FFM}D_t^{\psi ,\eta }} \alpha ) }{v_i}\nonumber \\ {_0^{FFM}D_t^{\psi ,\eta }} [{_0^{FFM}D_t^{\psi ,\eta }} J]= & {} - \gamma _i ({_0^{FFM}D_t^{\psi ,\eta }} \alpha )G - \gamma _i \alpha ({_0^{FFM}D_t^{\psi ,\eta }} G) + \gamma _i ({_0^{FFM}D_t^{\psi ,\eta }} \alpha )G_t - \mu _i ({_0^{FFM}D_t^{\psi ,\eta }} J) \end{aligned}$$

where

58 $$\begin{aligned} \Phi = (\alpha G +1) (\delta I ({_0^{FFM}D_t^{\psi ,\eta }} G) + \delta ({_0^{FFM}D_t^{\psi ,\eta }} I) G) - \delta I G (({_0^{FFM}D_t^{\psi ,\eta }} \alpha ) G + \alpha ({_0^{FFM}D_t^{\psi ,\eta }} G)) \end{aligned}$$

then we have

59 $$\begin{aligned} _0^{FFM}D_t^{\psi ,\eta }[_0^{FFM}D_t^{\psi ,\eta }] M\le & {} \Pi (G,I,\beta ,\alpha ,J) + \left( 1 + \frac{G^*}{G} \right) \{- b ({_0^{FFM}D_t^{\psi ,\eta }} G) + \delta _i ({_0^{FFM}D_t^{\psi ,\eta }} J) - \frac{\Phi }{(\alpha G + 1)^2}\}\nonumber \\+ & {} \left( 1 + \frac{I^*}{I} \right) \{\frac{(e)(\gamma ({_0^{FFM}D_t^{\psi ,\eta }} \beta )G^2 + 2 \gamma \beta G ({_0^{FFM}D_t^{\psi ,\eta }} G)) + \gamma ({_0^{FFM}D_t^{\psi ,\eta }} \beta ) G^4}{(e + G^2)^2} \nonumber \\- & {} \mu ({_0^{FFM}D_t^{\psi ,\eta }} I)\} + \left( 1 + \frac{\beta ^*}{\beta } \right) \left\{ \rho ({_0^{FFM}D_t^{\psi ,\eta }} \beta ) - \frac{2 \rho \beta ({_0^{FFM}D_t^{\psi ,\eta }} \beta ) }{v} \right\} \nonumber \\+ & {} \left( 1 + \frac{\alpha ^*}{\alpha } \right) \left\{ \rho _i ({_0^{FFM}D_t^{\psi ,\eta }} \alpha ) - \frac{2 \rho _i \alpha ({_0^{FFM}D_t^{\psi ,\eta }} \alpha ) }{v_i}\right\} + \left( 1 + \frac{J^*}{J} \right) \nonumber \\\times & {} \{ - \gamma _i ({_0^{FFM}D_t^{\psi ,\eta }} \alpha )G - \gamma _i \alpha ({_0^{FFM}D_t^{\psi ,\eta }} G) + \gamma _i ({_0^{FFM}D_t^{\psi ,\eta }} \alpha )G_t - \mu _i ({_0^{FFM}D_t^{\psi ,\eta }} J)\} \end{aligned}$$

where

60 $$\begin{aligned} \Pi (G,I,\beta ,\alpha ,J) = \left( \frac{_0^{FFM}D_t^{\psi ,\eta } G}{G} \right) ^2 G^* + \left( \frac{_0^{FFM}D_t^{\psi ,\eta } I}{I} \right) ^2 I^* + \left( \frac{_0^{FFM}D_t^{\psi ,\eta } \beta }{\beta } \right) ^2 \beta ^* + \left( \frac{_0^{FFM}D_t^{\psi ,\eta } \alpha }{\alpha } \right) ^2 \alpha ^*\nonumber \\ + \left( \frac{_0^{FFM}D_t^{\psi ,\eta } J}{J} \right) ^2 J^* \end{aligned}$$

now replacing ${_0^{FFM}D_t^{\psi ,\eta } G}, {_0^{FFM}D_t^{\psi ,\eta } I}, {_0^{FFM}D_t^{\psi ,\eta } \beta }, {_0^{FFM}D_t^{\psi ,\eta } \alpha }, {_0^{FFM}D_t^{\psi ,\eta } J}$ with their respective formula from the proposed model (7), we can get

61 $$\begin{aligned} {_0^{FFM}D_t^{\psi ,\eta }[_0^{FFM}D_t^{\psi ,\eta }] M} \le \Psi _1 + \Psi _2 \end{aligned}$$

$\Psi _1$: represents all positive terms

$\Psi _2$: represents all positive terms So that

- If $$\Psi _1 > \Psi _2$$ then $$ {_0^{FFM}D_t^{\psi ,\eta }[^{FFM}D_t^{\psi ,\eta }] M} > 0$$
- If $$\Psi _1 < \Psi _2$$ then $$ {_0^{FFM}D_t^{\psi ,\eta }[^{FFM}D_t^{\psi ,\eta }] M} < 0$$
- If $$\Psi _1 = \Psi _2$$ then $$ {_0^{FFM}D_t^{\psi ,\eta }[^{FFM}D_t^{\psi ,\eta }] M} = 0$$

## Computational analysis with fractal fractional operator

In this section, By using Mittag-Leffler Kernel for diabetes model given in (7), we get the simplist form as follows.

62 $$\begin{aligned} _0^{FFM}D_t^{\psi ,\eta } G= & {} G_1(t,G,I,\beta , \alpha , J) \nonumber \\ _0^{FFM}D_t^{\psi ,\eta } I= & {} I_1(t,G,I,\beta , \alpha , J) \nonumber \\ _0^{FFM}D_t^{\psi ,\eta } \beta= & {} \beta _1(t,G,I,\beta , \alpha , J) \nonumber \\ _0^{FFM}D_t^{\psi ,\eta } \alpha= & {} \alpha _1(t,G,I,\beta , \alpha , J) \nonumber \\ _0^{FFM}D_t^{\psi ,\eta } J= & {} J_1(t,G,I,\beta , \alpha , J) \end{aligned}$$

where

63 $$\begin{aligned} G_1(t,G,I,\beta , \alpha , J)= & {} \omega - bG - \frac{\delta I G}{\alpha G + 1} + \delta _i J \nonumber \\ I_1(t,G,I,\beta , \alpha , J)= & {} \frac{\gamma \beta G^2}{e + G^2} - \mu I \nonumber \\ \beta _1(t,G,I,\beta , \alpha , J)= & {} \rho \beta \left( 1- \frac{\beta }{v}\right) \nonumber \\ \alpha _1(t,G,I,\beta , \alpha , J)= & {} \rho _i \alpha \left( 1- \frac{\alpha }{v_i}\right) \nonumber \\ J_1(t,G,I,\beta , \alpha , J)= & {} - \gamma _i \alpha (G - G_t) - \mu _i J \end{aligned}$$

With Mittag-Leffler kernel applying fractal-fractional integral , we get

64 $$\begin{aligned} G(t_\varphi + 1)= & {} G(0) + \frac{1-\psi }{AB(\psi )}t_\varphi ^{1-\eta } G_1(t_\varphi , G(t_\varphi ),I(t_\varphi ),\beta (t_\varphi ),\alpha (t_\varphi ), J(t_\varphi ))\nonumber \\+ & {} \frac{\psi }{AB(\psi ) \Gamma (\psi )} \sum _{q=2}^\varphi \int _{t_q}^{t_{q+1}} G_1(t,G,I,\beta ,\alpha , J)\zeta ^{1-\eta }(t_{\varphi +1} - \zeta )^{\psi -1} d\zeta \end{aligned}$$

65 $$\begin{aligned} I(t_\varphi + 1)= & {} I(0) + \frac{1-\psi }{AB(\psi )}t_\varphi ^{1-\eta } I_1(t_\varphi , G(t_\varphi ),I(t_\varphi ),\beta (t_\varphi ),\alpha (t_\varphi ), J(t_\varphi ))\nonumber \\+ & {} \frac{\psi }{AB(\psi ) \Gamma (\psi )} \sum _{q=2}^\varphi \int _{t_q}^{t_{q+1}} I_1(t,G,I,\beta ,\alpha , J)\zeta ^{1-\eta }(t_{\varphi +1} - \zeta )^{\psi -1} d\zeta \end{aligned}$$

66 $$\begin{aligned} \beta (t_\varphi + 1)= & {} \beta (0) + \frac{1-\psi }{AB(\psi )}t_\varphi ^{1-\eta } \beta _1(t_\varphi , G(t_\varphi ),I(t_\varphi ),\beta (t_\varphi ),\alpha (t_\varphi ), J(t_\varphi ))\nonumber \\+ & {} \frac{\psi }{AB(\psi ) \Gamma (\psi )} \sum _{q=2}^\varphi \int _{t_q}^{t_{q+1}} \beta _1(t,G,I,\beta ,\alpha , J)\zeta ^{1-\eta }(t_{\varphi +1} - \zeta )^{\psi -1} d\zeta \end{aligned}$$

67 $$\begin{aligned} \alpha (t_\varphi + 1)= & {} \alpha (0) + \frac{1-\psi }{AB(\psi )}t_\varphi ^{1-\eta } \alpha _1(t_\varphi , G(t_\varphi ),I(t_\varphi ),\beta (t_\varphi ),\alpha (t_\varphi ), J(t_\varphi ))\nonumber \\+ & {} \frac{\psi }{AB(\psi ) \Gamma (\psi )} \sum _{q=2}^\varphi \int _{t_q}^{t_{q+1}} \alpha _1(t,G,I,\beta ,\alpha , J)\zeta ^{1-\eta }(t_{\varphi +1} - \zeta )^{\psi -1} d\zeta \end{aligned}$$

68 $$\begin{aligned} J(t_\varphi + 1)= & {} J(0) + \frac{1-\psi }{AB(\psi )}t_\varphi ^{1-\eta } J_1(t_\varphi , G(t_\varphi ),I(t_\varphi ),\beta (t_\varphi ),\alpha (t_\varphi ), J(t_\varphi ))\nonumber \\+ & {} \frac{\psi }{AB(\psi ) \Gamma (\psi )} \sum _{q=2}^\varphi \int _{t_q}^{t_{q+1}} J_1(t,G,I,\beta ,\alpha , J)\zeta ^{1-\eta }(t_{\varphi +1} - \zeta )^{\psi -1} d\zeta \end{aligned}$$

Recall the Newton Polynomial:

69 $$\begin{aligned} Q(t,G,I,\beta , \alpha , J)\simeq & {} Q(t_{\varphi -2}, G_{\varphi -2}, I_{\varphi -2}, \beta _{\varphi -2}, \alpha _{\varphi -2}, J_{\varphi -2}) + \frac{1}{\Delta t} [ Q(t_{\varphi -1}, G_{\varphi -1}, I_{\varphi -1}, \beta _{\varphi -1}, \alpha _{\varphi -1}, J_{\varphi -1})\nonumber \\- & {} Q(t_{\varphi -2}, G_{\varphi -2}, I_{\varphi -2}, \beta _{\varphi -2}, \alpha _{\varphi -2}, J_{\varphi -2})](\zeta - t_{\varphi -2}) +\frac{1}{2 \Delta t^2} [ Q(t_{\varphi }, G_{\varphi }, I_{\varphi }, \beta _{\varphi }, \alpha _{\varphi }, J_{\varphi })\nonumber \\- & {} 2 Q(t_{\varphi -1}, G_{\varphi -1}, I_{\varphi -1}, \beta _{\varphi -1}, \alpha _{\varphi -1}, J_{\varphi -1}) + Q(t_{\varphi -2}, G_{\varphi -2}, I_{\varphi -2}, \beta _{\varphi -2}, \alpha _{\varphi -2}, J_{\varphi -2})]\nonumber \\\times & {} (\zeta - t_{\varphi -2})(\zeta - t_{\varphi -1}) \end{aligned}$$

Replacing the Newton polynomial (69) into Eqs. (64)–(68), we have

70 $$\begin{aligned} G(t_\varphi + 1)= & {} G(0) + \frac{1-\psi }{AB(\psi )}t_\varphi ^{1-\eta } G_1(t_\varphi , G(t_\varphi ),I(t_\varphi ),\beta (t_\varphi ),\alpha (t_\varphi ),J(t_\varphi ))\nonumber \\+ & {} \frac{\psi }{AB(\psi ) \Gamma (\psi )} \sum _{q=2}^\varphi G_1(t_{q-2},G^{q-2},I^{q-2},\beta ^{q-2},\alpha ^{q-2},I^{q-2})t_{q-2}^{1-\eta }\int _{t_q}^{t_{q+1}}(t_{\varphi +1} - \zeta )^{\psi -1} d\zeta \nonumber \\+ & {} \frac{\psi }{AB(\psi ) \Gamma (\psi )} \sum _{q=2}^\varphi \frac{1}{\Delta t} \{ t_{q-1}^{1-\eta } G_1(t_{q-1},G^{q-1},I^{q-1},\beta ^{q-1},\alpha ^{q-1},J^{q-1}) - t_{q-2}^{1-\eta } \nonumber \\\times & {} G_1(t_{q-2},G^{q-2},I^{q-2},\beta ^{q-2},\alpha ^{q-2},J^{q-2}) \} \int _{t_q}^{t_{q+1}} (\zeta - t_{q-2})(t_{\varphi +1} - \zeta )^{\psi -1} d\zeta + \frac{\psi }{AB(\psi ) \Gamma (\psi )}\nonumber \\\times & {} \sum _{q=2}^\varphi \frac{1}{2 \Delta t^2} \{ t_{q}^{1-\eta } G_1(t_{q},G^{q},I^{q},\beta ^{q},\alpha ^{q},J^{q})-2 t_{q-1}^{1-\eta } G_1(t_{q-1},G^{q-1},I^{q-1},\beta ^{q-1},\alpha ^{q-1},J^{q-1}) \nonumber \\+ & {} t_{q-2}^{1-\eta } G_1(t_{q-2},G^{q-2},I^{q-2},\beta ^{q-2},\alpha ^{q-2},J^{q-2}) \} \int _{t_q}^{t_{q+1}} (\zeta - t_{q-2}) (\zeta - t_{q-1}) (t_{\varphi +1} - \zeta )^{\psi -1} d\zeta \end{aligned}$$

71 $$\begin{aligned} I(t_\varphi + 1)= & {} I(0) + \frac{1-\psi }{AB(\psi )}t_\varphi ^{1-\eta } I_1(t_\varphi , G(t_\varphi ),I(t_\varphi ),\beta (t_\varphi ),\alpha (t_\varphi ),J(t_\varphi ))\nonumber \\+ & {} \frac{\psi }{AB(\psi ) \Gamma (\psi )} \sum _{q=2}^\varphi I_1(t_{q-2},G^{q-2},I^{q-2},\beta ^{q-2},\alpha ^{q-2},I^{q-2})t_{q-2}^ {1-\eta }\int _{t_q}^{t_{q+1}}(t_{\varphi +1} - \zeta )^{\psi -1} d\zeta \nonumber \\+ & {} \frac{\psi }{AB(\psi ) \Gamma (\psi )} \sum _{q=2}^\varphi \frac{1}{\Delta t} \{ t_{q-1}^{1-\eta } I_1(t_{q-1},G^{q-1},I^{q-1},\beta ^{q-1},\alpha ^{q-1},J^{q-1}) - t_{q-2}^{1-\eta } \nonumber \\\times & {} I_1(t_{q-2},G^{q-2},I^{q-2},\beta ^{q-2},\alpha ^{q-2},J^{q-2}) \} \int _{t_q}^{t_{q+1}} (\zeta - t_{q -2})(t_{\varphi +1} - \zeta )^{\psi -1} d\zeta \frac{\psi }{AB(\psi ) \Gamma (\psi )}\nonumber \\\times & {} \sum _{q=2}^\varphi \frac{1}{2 \Delta t^2} \{ t_{q}^{1-\eta } I_1(t_{q},G^{q},I^{q},\beta ^{q},\alpha ^{q},J^{q})-2 t_{q-1}^{1-\eta } I_1(t_{q-1},G^{q-1},I^{q-1},\beta ^{q-1},\alpha ^{q-1},J^{q-1}) \nonumber \\+ & {} t_{q-2}^{1-\eta } I_1(t_{q-2},G^{q-2},I^{q-2},\beta ^{q-2},\alpha ^{q-2},J^{q-2}) \} \int _{t_q}^{t_{q+1}} (\zeta - t_{q-2}) (\zeta - t_{q-1}) (t_{\varphi +1} - \zeta )^{\psi -1} d\zeta \end{aligned}$$

72 $$\begin{aligned} \beta (t_\varphi + 1)= & {} \beta (0) + \frac{1-\psi }{AB(\psi )}t_\varphi ^{1-\eta } \beta _1(t_\varphi , G(t_\varphi ),I(t_\varphi ),\beta (t_\varphi ),\alpha (t_\varphi ),J(t_\varphi ))\nonumber \\+ & {} \frac{\psi }{AB(\psi ) \Gamma (\psi )} \sum _{q=2}^\varphi \beta _1(t_{q-2},G^{q-2},I^{q-2},\beta ^{q-2},\alpha ^{q-2},I^{q-2}) t_{q-2}^{1-\eta }\int _{t_q}^{t_{q+1}}(t_{\varphi +1} - \zeta )^{\psi -1} d\zeta \nonumber \\+ & {} \frac{\psi }{AB(\psi ) \Gamma (\psi )} \sum _{q=2}^\varphi \frac{1}{\Delta t} \{ t_{q-1}^{1-\eta } \beta _1(t_{q-1},G^{q-1},I^{q-1},\beta ^{q-1},\alpha ^{q-1},J^{q-1}) t_{q-2}^{1-\eta } \nonumber \\\times & {} \beta _1(t_{q-2},G^{q-2},I^{q-2},\beta ^{q-2},\alpha ^{q-2},J^{q-2}) \} \int _{t_q}^{t_{q+1}} (\zeta - t_{q-2})(t_{\varphi +1} - \zeta )^{\psi -1} d\zeta \frac{\psi }{AB(\psi ) \Gamma (\psi )} \nonumber \\\times & {} \sum _{q=2}^\varphi \frac{1}{2 \Delta t^2} \{ t_{q}^{1-\eta } \beta _1(t_{q},G^{q},I^{q},\beta ^{q},\alpha ^{q},J^{q})-2 t_{q-1}^{1-\eta } \beta _1(t_{q-1},G^{q-1},I^{q-1},\beta ^{q-1},\alpha ^{q-1},J^{q-1}) \nonumber \\+ & {} t_{q-2}^{1-\eta } \beta _1(t_{q-2},G^{q-2},I^{q-2},\beta ^{q-2},\alpha ^{q-2},J^{q-2}) \} \int _{t_q}^{t_{q+1}} (\zeta - t_{q-2}) (\zeta - t_{q-1}) (t_{\varphi +1} - \zeta )^{\psi -1} d\zeta \end{aligned}$$

73 $$\begin{aligned} \alpha (t_\varphi + 1)= & {} \alpha (0) + \frac{1-\psi }{AB(\psi )}t_\varphi ^{1-\eta } \alpha _1(t_\varphi , G(t_\varphi ),I(t_\varphi ),\beta (t_\varphi ),\alpha (t_\varphi ),J(t_\varphi ))\nonumber \\+ & {} \frac{\psi }{AB(\psi ) \Gamma (\psi )} \sum _{q=2}^\varphi \alpha _1(t_{q-2},G^{q-2},I^{q-2},\beta ^{q-2},\alpha ^{q-2},I^{q-2}) t_{q-2}^{1-\eta }\int _{t_q}^{t_{q+1}}(t_{\varphi +1} - \zeta )^{\psi -1} d\zeta \nonumber \\+ & {} \frac{\psi }{AB(\psi ) \Gamma (\psi )} \sum _{q=2}^\varphi \frac{1}{\Delta t} \{ t_{q-1}^{1-\eta } \alpha _1(t_{q-1},G^{q-1},I^{q-1},\beta ^{q-1},\alpha ^{q-1},J^{q-1}) t_{q-2}^{1-\eta } \nonumber \\\times & {} \alpha _1(t_{q-2},G^{q-2},I^{q-2},\beta ^{q-2},\alpha ^{q-2},J^{q-2}) \} \int _{t_q}^{t_{q+1}} (\zeta - t_{q-2})(t_{\varphi +1} - \zeta )^{\psi -1} d\zeta \frac{\psi }{AB(\psi ) \Gamma (\psi )} \nonumber \\\times & {} \sum _{q=2}^\varphi \frac{1}{2 \Delta t^2} \{ t_{q}^{1-\eta } \alpha _1(t_{q},G^{q},I^{q},\beta ^{q},\alpha ^{q},J^{q})-2 t_{q-1}^{1-\eta } \alpha _1 (t_{q-1},G^{q-1},I^{q-1},\beta ^{q-1},\alpha ^{q-1},J^{q-1}) \nonumber \\+ & {} t_{q-2}^{1-\eta } \alpha _1(t_{q-2},G^{q-2},I^{q-2},\beta ^{q-2},\alpha ^{q-2},J^{q-2}) \} \int _{t_q}^{t_{q+1}} (\zeta - t_{q-2}) (\zeta - t_{q-1}) (t_{\varphi +1} - \zeta )^{\psi -1} d\zeta \end{aligned}$$

74 $$\begin{aligned} J(t_\varphi + 1)= & {} J(0) + \frac{1-\psi }{AB(\psi )}t_\varphi ^{1-\eta } J_1 (t_\varphi , G(t_\varphi ),I(t_\varphi ),\beta (t_\varphi ), \alpha (t_\varphi ),J(t_\varphi ))\nonumber \\+ & {} \frac{\psi }{AB(\psi ) \Gamma (\psi )} \sum _{q=2}^\varphi J_1(t_{q-2},G^{q-2},I^{q-2},\beta ^{q-2},\alpha ^{q-2},I^{q-2}) t_{q-2}^{1-\eta }\int _{t_q}^{t_{q+1}}(t_{\varphi +1} - \zeta )^{\psi -1} d\zeta \nonumber \\+ & {} \frac{\psi }{AB(\psi ) \Gamma (\psi )} \sum _{q=2}^\varphi \frac{1}{\Delta t} \{ t_{q-1}^{1-\eta } J_1(t_{q-1},G^{q-1},I^{q-1},\beta ^{q-1},\alpha ^{q-1},J^{q-1}) t_{q-2}^{1-\eta } \nonumber \\\times & {} J_1(t_{q-2},G^{q-2},I^{q-2},\beta ^{q-2},\alpha ^{q-2},J^{q-2}) \} \int _{t_q}^{t_{q+1}} (\zeta - t_{q-2})(t_{\varphi +1} - \zeta )^{\psi -1} d\zeta \frac{\psi }{AB(\psi ) \Gamma (\psi )} \nonumber \\\times & {} \sum _{q=2}^\varphi \frac{1}{2 \Delta t^2} \{ t_{q}^{1-\eta } J_1(t_{q},G^{q},I^{q},\beta ^{q},\alpha ^{q},J^{q})-2 t_{q-1}^{1-\eta } J_1(t_{q-1},G^{q-1},I^{q-1},\beta ^{q-1},\alpha ^{q-1},J^{q-1}) \nonumber \\+ & {} t_{q-2}^{1-\eta } J_1(t_{q-2},G^{q-2},I^{q-2},\beta ^{q-2},\alpha ^{q-2},J^{q-2}) \} \int _{t_q}^{t_{q+1}} (\zeta - t_{q-2}) (\zeta - t_{q-1}) (t_{\varphi +1} - \zeta )^{\psi -1} d\zeta \end{aligned}$$

Calculations for the integral in the Eqs. (70)–(74) are:

75 $$\begin{aligned} \int _{t_q}^{t_{q+1}}(t_{\varphi +1} - \zeta )^{\psi -1} d\zeta= & {} \frac{(\Delta t)^\psi }{\psi } \left( (\varphi - q + 1)^\psi - (\varphi - q)^\psi \right) \end{aligned}$$

76 $$\begin{aligned} \int _{t_q}^{t_{q+1}} (\zeta - t_{q-2})(t_{\varphi +1} - \zeta )^{\psi -1} d\zeta= & {} \frac{(\Delta t)^{\psi +1}}{\psi (\psi +1)} \{ (\varphi - q + 1)^\psi (\varphi -q +3 +2\psi )\nonumber \\- & {} (\varphi - q)^\psi (\varphi - q +3 +3\psi ) \} \end{aligned}$$

77 $$\begin{aligned} \int _{t_q}^{t_{q+1}} (\zeta - t_{q -2}) (\zeta - t_{q-1}) (t_{\varphi +1} - \zeta )^{\psi -1} d\zeta= & {} \frac{(\Delta t)^{\psi +2}}{\psi (\psi +1)(\psi +2)} \{ (\varphi - q + 1)^\psi (2(\varphi -q)^2\nonumber \\+ & {} (3\psi +10)(\varphi - q) +2\psi ^2 +9\psi +12) - (\varphi - q)^\psi \nonumber \\\times & {} (2(\varphi - q)^2 +(5\psi + 10)(\varphi - q) + 6 \psi ^2 +18\psi +12) \} \end{aligned}$$

Hence, we get finally

78 $$\begin{aligned} G(t_\varphi + 1)= & {} G(0) + \frac{1-\psi }{AB(\psi )}t_\varphi ^{1-\eta } G_1(t_\varphi ,G(t_\varphi ),I(t_\varphi ),\beta (t_\varphi ),\alpha (t_\varphi ),J(t_\varphi ))\nonumber \\+ & {} \frac{\psi (\Delta t)^\psi }{AB(\psi ) \Gamma (\psi +1)} \sum _{q=2}^\varphi G_1(t_{q-2},G^{q-2},I^{q-2},\beta ^{q-2},\alpha ^{q-2},J^{q-2})t_{q-2}^{1-\eta }\left( (\varphi - q + 1)^\psi - (\varphi - q)^\psi \right) \nonumber \\+ & {} \frac{\psi (\Delta t)^\psi }{AB(\psi ) \Gamma (\psi +2)} \sum _{q=2}^\varphi \{ t_{q-1}^{1-\eta } G_1(t_{q-1},G^{q-1},I^{q-1},\beta ^{q-1},\alpha ^{q-1},J^{q-1})- t_{q-2}^{1-\eta }\nonumber \\\times & {} G_1(t_{q-2},G^{q-2},I^{q-2},\beta ^{q-2},\alpha ^{q-2},J^{q-2}) \}\{ (\varphi - q + 1)^\psi (\varphi -q +3 +2\psi ) - (\varphi - q)^\psi \nonumber \\\times & {} (\varphi - q +3 +3\psi ) \} + \frac{\psi (\Delta t)^\psi }{2AB(\psi ) \Gamma (\psi +3)} \sum _{q=2}^\varphi \{ t_{q}^{1-\eta } G_1(t_{q},G^{q},I^{q},\beta ^{q},\alpha ^{q},J^{q})-2 t_{q-1}^{1-\eta } \nonumber \\\times & {} G_1(t_{q-1},G^{q-1},I^{q-1},\beta ^{q-1},\alpha ^{q-1},J^{q-1}) + t_{q-2}^{1-\eta } G_1(t_{q-2},G^{q-2},I^{q-2},\beta ^{q-2},\alpha ^{q-2},J^{q-2}) \} \nonumber \\\times & {} \{ (\varphi - q + 1)^\psi (2(\varphi -q)^2 (3\psi +10)(\varphi - q) +2\psi ^2 + 9\psi +12)- (\varphi - q)^\psi (2(\varphi - q)^2\nonumber \\+ & {} (5\psi + 10)(\varphi - q) + 6 \psi ^2 +18\psi +12) \} \end{aligned}$$

79 $$\begin{aligned} I(t_\varphi + 1)= & {} I(0) + \frac{1-\psi }{AB(\psi )}t_\varphi ^{1-\eta } I_1(t_\varphi ,G(t_\varphi ),I(t_\varphi ),\beta (t_\varphi ),\alpha (t_\varphi ),J(t_\varphi ))\nonumber \\+ & {} \frac{\psi (\Delta t)^\psi }{AB(\psi ) \Gamma (\psi +1)} \sum _{q=2}^\varphi I_1(t_{q-2},G^{q-2},I^{q-2},\beta ^{q-2},\alpha ^{q-2},J^{q-2})t_{q-2}^{1-\eta }\left( (\varphi - q + 1)^\psi - (\varphi - q)^\psi \right) \nonumber \\+ & {} \frac{\psi (\Delta t)^\psi }{AB(\psi ) \Gamma (\psi +2)} \sum _{q=2}^\varphi \{ t_{q-1}^{1-\eta } I_1(t_{q-1},G^{q-1},I^{q-1},\beta ^{q-1},\alpha ^{q-1},J^{q-1})- t_{q-2}^{1-\eta }\nonumber \\\times & {} I_1(t_{q-2},G^{q-2},I^{q-2},\beta ^{q-2},\alpha ^{q-2},J^{q-2}) \}\{ (\varphi - q + 1)^\psi (\varphi -q +3 +2\psi ) - (\varphi - q)^\psi \nonumber \\\times & {} (\varphi - q +3 +3\psi ) \} + \frac{\psi (\Delta t)^\psi }{2AB(\psi ) \Gamma (\psi +3)} \sum _{q=2}^\varphi \{ t_{q}^{1-\eta } I_1(t_{q},G^{q},I^{q},\beta ^{q},\alpha ^{q},J^{q})-2 t_{q-1}^{1-\eta } \nonumber \\\times & {} I_1(t_{q-1},G^{q-1},I^{q-1},\beta ^{q-1},\alpha ^{q-1},J^{q-1}) + t_{q-2}^{1-\eta } I_1(t_{q-2},G^{q-2},I^{q-2},\beta ^{q-2},\alpha ^{q-2},J^{q-2}) \} \nonumber \\\times & {} \{ (\varphi - q + 1)^\psi (2(\varphi -q)^2 (3\psi +10)(\varphi - q) +2\psi ^2 + 9\psi +12)- (\varphi - q)^\psi (2(\varphi - q)^2\nonumber \\+ & {} (5\psi + 10)(\varphi - q) + 6 \psi ^2 +18\psi +12) \} \end{aligned}$$

80 $$\begin{aligned} \beta (t_\varphi + 1)= & {} \beta (0) + \frac{1-\psi }{AB(\psi )}t_\varphi ^{1-\eta } \beta _1(t_\varphi ,G(t_\varphi ),I(t_\varphi ),\beta (t_\varphi ), \alpha (t_\varphi ),J(t_\varphi ))\nonumber \\+ & {} \frac{\psi (\Delta t)^\psi }{AB(\psi ) \Gamma (\psi +1)} \sum _{q=2}^\varphi \beta _1(t_{q-2},G^{q-2},I^{q-2},\beta ^{q-2},\alpha ^{q-2},J^{q-2})t_{q-2}^{1-\eta }\left( (\varphi - q + 1)^\psi - (\varphi - q)^\psi \right) \nonumber \\+ & {} \frac{\psi (\Delta t)^\psi }{AB(\psi ) \Gamma (\psi +2)} \sum _{q=2}^\varphi \{ t_{q-1}^{1-\eta } \beta _1(t_{q-1},G^{q-1},I^{q-1},\beta ^{q-1},\alpha ^{q-1},J^{q-1})- t_{q-2}^{1-\eta }\nonumber \\\times & {} \beta _1(t_{q-2},G^{q-2},I^{q-2},\beta ^{q-2},\alpha ^{q-2},J^{q-2}) \}\{ (\varphi - q + 1)^\psi (\varphi -q +3 +2\psi ) - (\varphi - q)^\psi \nonumber \\\times & {} (\varphi - q +3 +3\psi ) \} + \frac{\psi (\Delta t)^\psi }{2AB(\psi ) \Gamma (\psi +3)} \sum _{q=2}^\varphi \{ t_{q}^{1-\eta } \beta _1(t_{q},G^{q},I^{q},\beta ^{q},\alpha ^{q},J^{q})-2 t_{q-1}^{1-\eta } \nonumber \\\times & {} \beta _1(t_{q-1},G^{q-1},I^{q-1},\beta ^{q-1},\alpha ^{q-1},J^{q-1}) + t_{q-2}^{1-\eta } \beta _1(t_{q-2},G^{q-2},I^{q-2},\beta ^{q-2},\alpha ^{q-2},J^{q-2}) \} \nonumber \\\times & {} \{ (\varphi - q + 1)^\psi (2(\varphi -q)^2 (3\psi +10)(\varphi - q) +2\psi ^2 + 9\psi +12)- (\varphi - q)^\psi (2(\varphi - q)^2\nonumber \\+ & {} (5\psi + 10)(\varphi - q) + 6 \psi ^2 +18\psi +12) \} \end{aligned}$$

81 $$\begin{aligned} \alpha (t_\varphi + 1)= & {} \alpha (0) + \frac{1-\psi }{AB(\psi )}t_\varphi ^{1-\eta } \alpha _1(t_\varphi ,G(t_\varphi ),I(t_\varphi ), \beta (t_\varphi ),\alpha (t_\varphi ),J(t_\varphi ))\nonumber \\+ & {} \frac{\psi (\Delta t)^\psi }{AB(\psi ) \Gamma (\psi +1)} \sum _{q=2}^\varphi \alpha _1(t_{q-2},G^{q-2},I^{q-2},\beta ^{q-2},\alpha ^{q-2},J^{q-2})t_{q-2}^{1-\eta }\left( (\varphi - q + 1)^\psi - (\varphi - q)^\psi \right) \nonumber \\+ & {} \frac{\psi (\Delta t)^\psi }{AB(\psi ) \Gamma (\psi +2)} \sum _{q=2}^\varphi \{ t_{q-1}^{1-\eta } \alpha _1(t_{q-1},G^{q-1},I^{q-1},\beta ^{q-1},\alpha ^{q-1},J^{q-1})- t_{q-2}^{1-\eta }\nonumber \\\times & {} \alpha _1(t_{q-2},G^{q-2},I^{q-2},\beta ^{q-2},\alpha ^{q-2},J^{q-2}) \}\{ (\varphi - q + 1)^\psi (\varphi -q +3 +2\psi ) - (\varphi - q)^\psi \nonumber \\\times & {} (\varphi - q +3 +3\psi ) \} + \frac{\psi (\Delta t)^\psi }{2AB(\psi ) \Gamma (\psi +3)} \sum _{q=2}^\varphi \{ t_{q}^{1-\eta } \alpha _1(t_{q},G^{q},I^{q},\beta ^{q},\alpha ^{q},J^{q})-2 t_{q-1}^{1-\eta } \nonumber \\\times & {} \alpha _1(t_{q-1},G^{q-1},I^{q-1},\beta ^{q-1},\alpha ^{q-1},J^{q-1}) + t_{q-2}^{1-\eta } \alpha _1(t_{q-2},G^{q-2},I^{q-2},\beta ^{q-2},\alpha ^{q-2},J^{q-2}) \} \nonumber \\\times & {} \{ (\varphi - q + 1)^\psi (2(\varphi -q)^2 (3\psi +10)(\varphi - q) +2\psi ^2 + 9\psi +12)- (\varphi - q)^\psi (2(\varphi - q)^2\nonumber \\+ & {} (5\psi + 10)(\varphi - q) + 6 \psi ^2 +18\psi +12) \} \end{aligned}$$

82 $$\begin{aligned} J(t_\varphi + 1)= & {} J(0) + \frac{1-\psi }{AB(\psi )}t_\varphi ^{1-\eta } J_1(t_\varphi ,G(t_\varphi ),I(t_\varphi ),\beta (t_\varphi ),\alpha (t_\varphi ),J(t_\varphi ))\nonumber \\+ & {} \frac{\psi (\Delta t)^\psi }{AB(\psi ) \Gamma (\psi +1)} \sum _{q=2}^\varphi J_1(t_{q-2},G^{q-2},I^{q-2},\beta ^{q-2},\alpha ^{q-2},J^{q-2})t_{q-2}^{1-\eta }\left( (\varphi - q + 1)^\psi - (\varphi - q)^\psi \right) \nonumber \\+ & {} \frac{\psi (\Delta t)^\psi }{AB(\psi ) \Gamma (\psi +2)} \sum _{q=2}^\varphi \{ t_{q-1}^{1-\eta } J_1(t_{q-1},G^{q-1},I^{q-1},\beta ^{q-1},\alpha ^{q-1},J^{q-1})- t_{q-2}^{1-\eta }\nonumber \\\times & {} J_1(t_{q-2},G^{q-2},I^{q-2},\beta ^{q-2},\alpha ^{q-2},J^{q-2}) \}\{ (\varphi - q + 1)^\psi (\varphi -q +3 +2\psi ) - (\varphi - q)^\psi \nonumber \\\times & {} (\varphi - q +3 +3\psi ) \} + \frac{\psi (\Delta t)^\psi }{2AB(\psi ) \Gamma (\psi +3)} \sum _{q=2}^\varphi \{ t_{q}^{1-\eta } J_1(t_{q},G^{q},I^{q},\beta ^{q},\alpha ^{q},J^{q})-2 t_{q-1}^{1-\eta } \nonumber \\\times & {} J_1(t_{q-1},G^{q-1},I^{q-1},\beta ^{q-1},\alpha ^{q-1},J^{q-1}) + t_{q-2}^{1-\eta } J_1(t_{q-2},G^{q-2},I^{q-2},\beta ^{q-2},\alpha ^{q-2},J^{q-2}) \} \nonumber \\\times & {} \{ (\varphi - q + 1)^\psi (2(\varphi -q)^2 (3\psi +10)(\varphi - q) +2\psi ^2 + 9\psi +12)- (\varphi - q)^\psi (2(\varphi - q)^2\nonumber \\+ & {} (5\psi + 10)(\varphi - q) + 6 \psi ^2 +18\psi +12) \} \end{aligned}$$

## Numerical simulation and discussion

In this section, the numerical simulation of the proposed method using the mittage-leffler kernel for the diabetes model is discussed. The system’s parameter values and initial conditions^8^ are listed in table 1 that is given below

**Table 1:** Parameter values and biological interpretation.

Parameters	Biological interpretation	Values	Units
$\omega$	Production rate of glucose for $G=0$	864	mg/(dl $\cdot$ d)
*b*	Glucose clearance rate independent of insulin	1.44	d$^{-1}$
$\delta$	Glucose uptake rate induced by insulin	0.85	ml/(mU $\cdot$ d)
$\delta _i$	Glucose production rate induced by glucagon	1350	d$^{-1}$
$\gamma$	The maximum insulin secretory rate by $\beta$-cell	43.2	mU/(ml $\cdot$ d $\cdot$ mg)
$\gamma _i$	The maximum glucagon secretory rate by $\alpha$-cell	0.05	1/(mg $\cdot$ d)
*e*	Inflection point for sigmoid	20000	mg$^2$/dl$^2$
$\mu$	Insulin clearance rate for complete body	432	d$^{-1}$
$\mu _i$	Glucagon clearance rate for complete body	0.3	d$^{-1}$
*v*	Environmental capacity of $\beta$-cell mass	900	mg
$v_i$	$\alpha$-cell mass environmental capacity	300	mg
$\alpha$	Half saturation inverse as a constant	0.01	mg
$G_t$	Glycaemia minimum level	80	mg/dl

**Figure 1:**
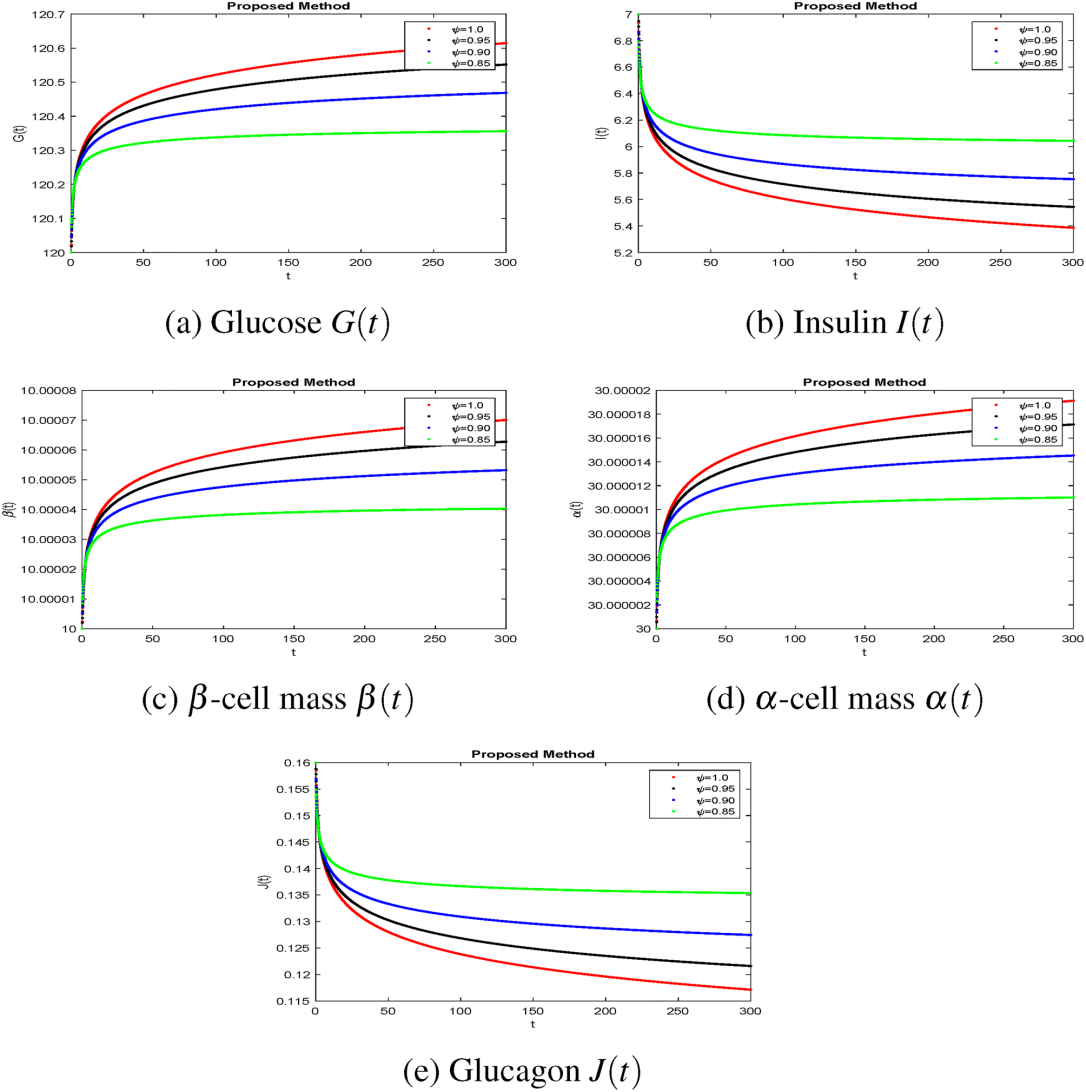
Simulation of the diabetes model compartments at initial condition (120, 07, 10, 30, 0.16).

**Figure 2:**
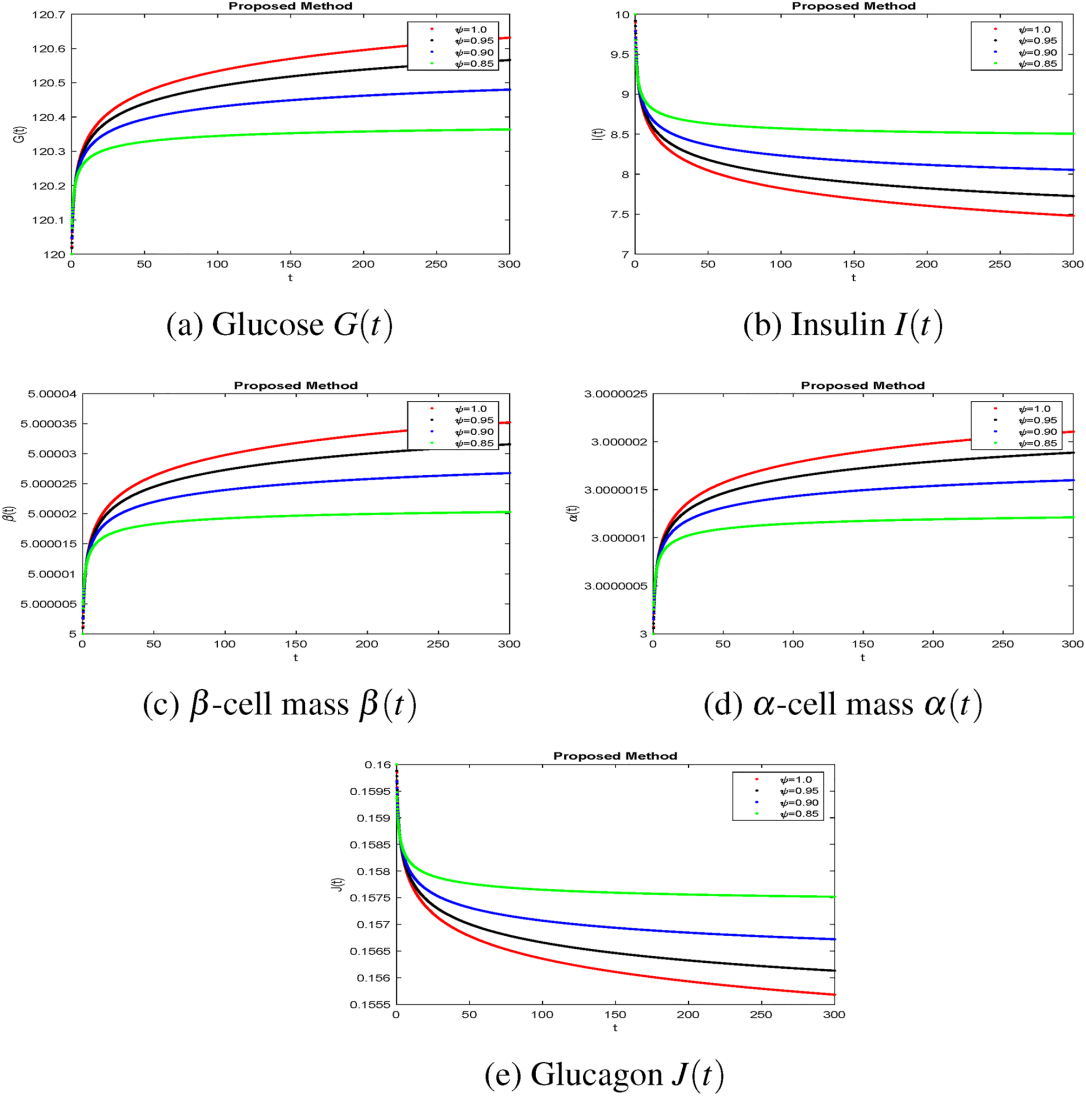
Simulation of the diabetes model compartments at initial condition (120, 10, 05, 03, 0.16).

**Figure 3:**
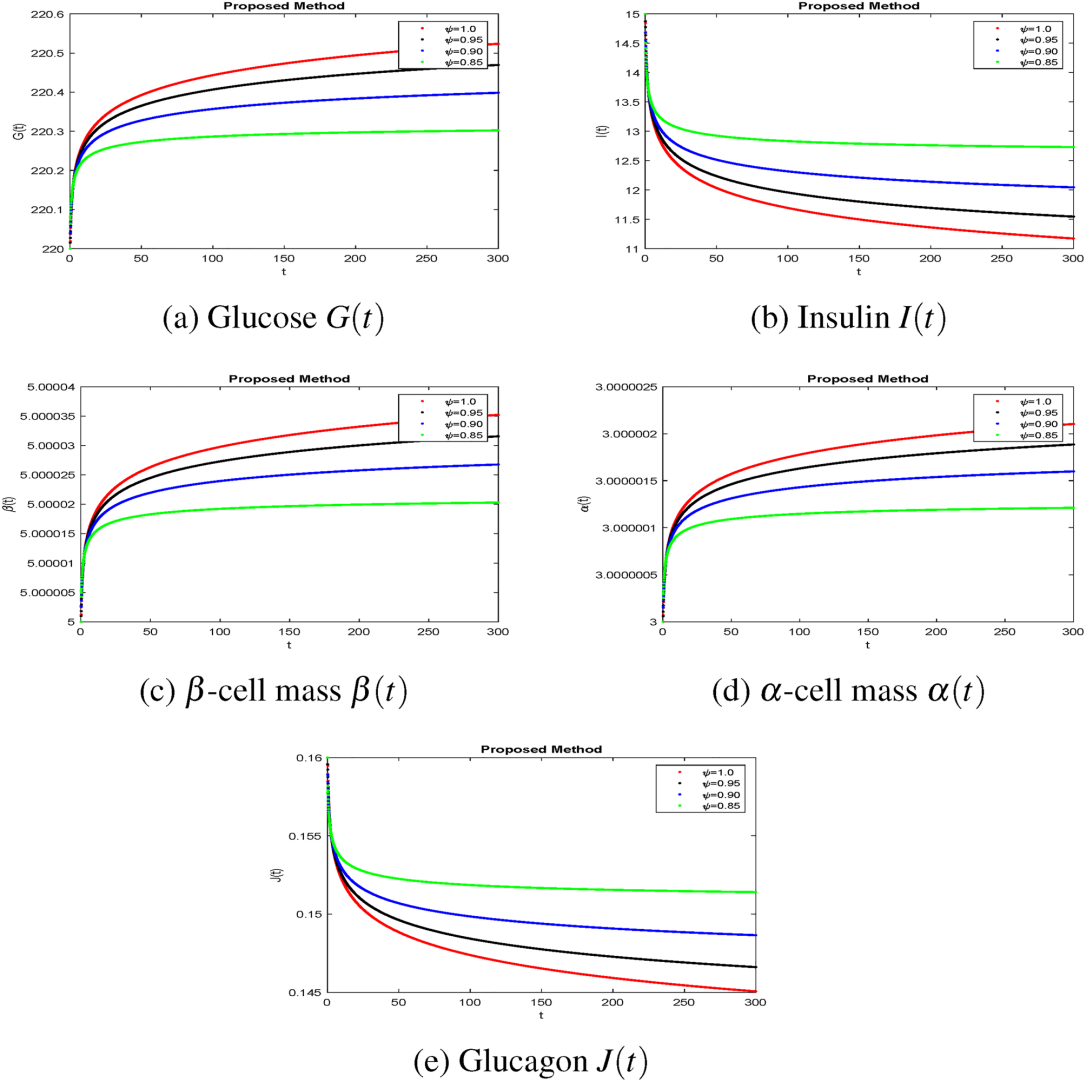
Simulation of the diabetes model compartments at initial condition (220, 15, 05, 03, 0.16).

The effectiveness of the obtained theoretical outcomes is established by using advanced techniques. The mathematical analysis of diabetes with hormonal effects is analysed through simulation in Figs. 1, 2, 3 at different initial conditions. In Figs. 1, 2, 3, solutions for all compartments are shown with different fractional order values at a fixed fractal dimension of the system. Matlab coding is employed to find the numerical simulation for the fractional-order diabetes model. A range of values for $\psi (\psi = 0.85, 0.90, 0.95, 1)$ are shown to illustrate the dynamic effects. Interestingly, glucose, $\beta$ mass, and $\alpha$ mass decrease while insulin and glucagon increase when taking into account a fractional order. Still, there is a marked drop in gulcagon and insulin as $\psi$ gets closer to 1. The findings highlight how $\psi$ affects the system’s dynamics. The normal glucose concentration level in human blood is in a narrow range (80–180 mg/dl). In Fig. 1, glucose level, insulin level, and $\alpha -$ cell mass with low glucose concentrations in people with diabetes decrease by decreasing fractional values, while the $\beta -$ cell which produces insulin and glucagon, starts rising by decreasing fractional values. Similar behavior can be seen in Fig 2 with minor change in the initial condition. Similarly, by changing the initial condition again as a third case to be considered to see its behavior within the bounded domain. It is observed that glucose levels decrease due to the rise in insulin by $\beta -$ cells, which will be helpful for diabetes patients. Diabetes patients will approach a stable position due to a rise in insulin level and maintain it after a certain period. It also demonstrates that when there is hypoglycemia, the $\alpha$-cells secrete glucagon to keep the blood sugar levels within the normal range. This finding highlights the value of fractional calculus in explaining the intricate and persistent behaviour of the model and reveals the system’s innate stability and resilience. As such, the fractional dimension $\psi$ becomes crucial in the diabetes mellitus model simulation tests carried out in this work. It predicts what should happen in the future through this research and how we will be able to reduce the spread of diabetes in society. The fractal-fractional method provides reliable findings for all compartments according to steady state at non-integer order derivatives as compared to classical derivatives.

## Conclusion

In this work, the fractional order diabetes model is studied to impact inulin and glucagons for administrations of gulose in the human body. In this regard, qualitative and quantitative properties of the analysis, such as global stability, uniqueness of the solution, and positivity with fixed point theory, result. Results through figures are derived with the help of a fractal fractional operator utilising the Mittag-Leffler kernel, which provides us with continuous monitoring of the glucose-insulin relationship in the human body at different fractional order values. It is observed that maintaining the glucose level within the usual range is the major responsibility of the $\beta$ and $\alpha$ cells in the pancreas to produce the hormones insulin and glucagon, respectively. However, diabetes can result from $\beta$-cell and $\alpha$-cell malfunction. The research on how glucose, insulin, $\beta$-cells, $\alpha$-cells, and glucagon interact has been avoided. These results play a key role in the study of the glucose-insulin-glucagon relationship, which is helpful for close-loop design (artificial pancreas) to control type 1 diabetes. A closed-loop design for a glucose-insulin pump plays an important role in overcoming the risk of hypoglycemia and hyperglycemia in humans. Research in this area will advance as a result of this method’s improved understanding of the dynamics and behaviour of diabetes mellitus. In the future, we will analyse the prediction model for treating and controlling diabetes in society with novel and modified fractional operators. Using non-local and non-singular kernel operators, such as Caputo–Fabrizio and ABC differential (integral) operators, can better capture empirical events than traditional mathematical operators, leading to deeper insights into the diabetes mellitus model. These fractional operators can be used to represent the diabetes mellitus disease, and their relative advantages and disadvantages can be evaluated. It would therefore be beneficial for aspiring young researchers to compare their findings with the findings of this study.

## Data Availability

All data generated or analysed during this study are included in this Manuscript.
